# Evolutionarily Conserved but Mechanistically Distinct Mitochondrial Responses to Loss of Timeless/Swi1

**DOI:** 10.3390/biom16081177

**Published:** 2026-08-12

**Authors:** Kalisse I. Horne, Joshua Chang Mell, Chiaki Noguchi, Sri Havya Jana, Shriya Pinisetty, Rhea Masand, Christian Sell, Eishi Noguchi

**Affiliations:** 1 Program in Molecular and Cell Biology and Genetics, Graduate School of Biomedical Sciences and Professional Studies, Drexel University College of Medicine, Philadelphia, PA 19102, USA; kh3376@drexel.edu (K.I.H.); srihavyajana@gmail.com (S.H.J.); 2 Department of Biochemistry and Molecular Biology, Drexel University College of Medicine, Philadelphia, PA 19102, USA; xchiakinx@gmail.com (C.N.); sp3849@drexel.edu (S.P.); rm3697@drexel.edu (R.M.); cs389@drexel.edu (C.S.); 3 Department of Microbiology and Immunology, Drexel University College of Medicine, Philadelphia, PA 19102, USA; jcm385@drexel.edu

**Keywords:** Timeless, Swi1, fission yeast, *Schizosaccharomyces pombe*, mitochondria, mtDNA, DNA polymerase γ, stress response, mitochondrial genome, oxidative stress

## Abstract

Timeless and its fission yeast ortholog Swi1 are evolutionarily conserved components of the replication fork protection complex that ensures faithful DNA replication and genome stability. While their nuclear roles are well-characterized, their roles in mitochondrial genome maintenance remain unknown. Here, we demonstrate a previously unrecognized connection between Timeless/Swi1 and mitochondrial homeostasis. In fission yeast, *swi1* deletion increased association of the DNA repair protein Rad52 with mitochondrial DNA sequences across the mitochondrial genome, suggesting altered mitochondrial genome maintenance. Unexpectedly, *swi1*∆ cells showed an increased mtDNA copy number and improved growth under respiratory conditions, suggesting activation of compensatory mechanisms that promote mitochondrial genome maintenance. The loss of Swi1 also partially rescued the growth defect under respiratory conditions and mtDNA loss associated with depletion of mitochondrial DNA polymerase γ, linking Swi1 to pathways regulating mitochondrial replication under stress. Consistent with these phenotypes, transcriptomic and pathway enrichment analyses revealed transcriptional changes indicative of reduced glycolysis and enhanced oxidative phosphorylation, suggesting a shift toward respiratory metabolism. In human cells, Timeless depletion elicited distinct mitochondrial responses depending on the cell type. While Timeless-depleted TE-11 and Saos-2 cells elicited mitochondrial phenotypes comparable to those observed in fission yeast, Timeless depletion in U-2 OS cells led to reduced mtDNA copy number, elevated mitochondrial reactive oxygen species, and decreased mitochondrial membrane potential and mass, consistent with mitochondrial dysfunction. Despite these phenotypic differences, both fission yeast and human cells exhibited elevated levels of orthologs of the mitochondrial transcription factor A (TFAM) and the oxidative stress regulator NRF2, suggesting the conserved activation of compensatory mitochondrial and antioxidant pathways. Together, these findings identify an evolutionarily conserved connection between Timeless/Swi1 and mitochondrial homeostasis and reveal distinct adaptive responses to mitochondrial stress.

## 1. Introduction

Timeless is conserved amongst eukaryotes and plays a key role in DNA replication and genome stability. In both yeast and mammals, Timeless (Swi1 in the fission yeast *Schizosaccharomyces pombe*; Tof1 in the budding yeast *Saccharomyces cerevisiae*) functions as the major subunit of the replication fork protection complex that is critical for stabilizing replication forks, coordinating S-phase checkpoint responses, and preventing replication-transcription conflicts, thereby contributing to genome maintenance [1,2,3]. Consistent with its role in maintaining replication fork stability, the loss of Timeless/Swi1 causes replication stress and accumulation of DNA damage in the nuclear genome, particularly at difficult-to-replicate genomic regions, including telomeres, rDNA loci, repetitive DNA sequences, and ectopically introduced *LacO* repeat arrays [1,2,3,4,5].

While the nuclear functions of Timeless/Swi1 have been well-characterized, its potential role in mitochondrial function remains unknown. Mitochondria possess their own genome (mtDNA), which must be faithfully replicated and maintained to ensure proper oxidative phosphorylation and energy production. The loss of mitochondrial genome integrity is associated with aging, neurodegenerative diseases, and metabolic disorders [6,7,8]. Unlike nuclear DNA replication, mitochondrial DNA replication is carried out by DNA polymerase γ (Pol γ). Moreover, because mitochondria continuously generate reactive oxygen species (ROS) as a by-product of oxidative phosphorylation, leading to non-bulky base lesions, base excision repair is the primary DNA repair mechanism in mitochondria. However, studies indicate that homologous recombination also operates in this organelle [9]. In addition, studies have suggested that nuclear DNA repair and replication proteins can influence mitochondrial genome dynamics, either through direct localization or indirect regulatory mechanisms [10,11,12]. Rad52 is a key homologous recombination factor that binds single-stranded DNA and promotes recombination-mediated repair of damaged DNA. Studies have shown that members of the Rad52 epistasis group, including Rad51, Rad52, and Rad59, contribute to mitochondrial DNA repair and maintenance, and that some of these proteins localize to mitochondria in both yeast and human cells [13,14,15,16]. Furthermore, the mitochondrial DNA copy number is dynamically regulated in response to metabolic state, mitochondrial dysfunction, and cellular stress, suggesting that changes in mtDNA abundance may represent an adaptive response to compromised mitochondrial function [17].

In this study, we explore the role of Timeless/Swi1 in mitochondrial genome maintenance and function. We show that Swi1 deletion in *S. pombe* results in increased Rad52 association across the mitochondrial genome, consistent with elevated mitochondrial genome stress and instability, and is accompanied by a substantial increase in mtDNA copy number. Moreover, through genetic interaction studies with *pog1*, which encodes Pol γ in *S. pombe*, we demonstrate that Swi1 loss partially suppresses the growth defects caused by impaired mitochondrial DNA replication. In human cells, mitochondrial responses are cell-type-dependent. Timeless depletion in TE-11 esophageal cancer cells and Saos-2 osteosarcoma cells results in mitochondrial phenotypes similar to those observed in fission yeast; however, its depletion in U-2 OS cells leads to a reduced mtDNA copy number, elevated mitochondrial reactive oxygen species (mtROS), and decreased mitochondrial membrane potential, consistent with mitochondrial dysfunction. Despite these cell-type-specific differences, our findings support a previously unrecognized connection between Timeless/Swi1 and mitochondrial homeostasis. Together, our data uncover a novel function for Timeless/Swi1 in mitochondrial homeostasis and provide new insight into the coordination of nuclear and mitochondrial genome maintenance.

## 2. Materials and Methods

### 2.1. General Techniques and Strains Used in S. pombe Studies

The *S. pombe* strains used in this study were constructed using standard genetic and molecular techniques [18], and their genotypes are listed in Table A1. Unless otherwise specified, *S. pombe* cells were grown in yeast extract and supplements (YES) medium (0.5% yeast extract and 3% glucose supplemented with adenine, histidine-HCL, leucine, and uracil) at 25 °C or 30 °C as previously described [18]. For experiments involving regulation of the thiamine-repressible *nmt81* promoter, cells were grown in Edinburgh minimal medium (EMM2) and supplemented as described [18]. For growth under respiration conditions, *S. pombe* cells were cultured in YEGlyE medium containing 1% yeast extract, 0.5% casamino acid, 3% glycerol, and 3% ethanol, and supplemented as indicated above.

For selection of *S. pombe* strains carrying specific antibiotic markers, G418 (for *kanMX6*), hygromycin B (for *hphMX6*), and nourseothricin (for *natMX6*) were used at final concentrations of 150 µg/mL, 100 µg/mL, and 100 µg/mL, respectively.

The *nmt81-pog1* (*nmt81-3FLAG-pog1*:*kanMX6*) allele was constructed by a two-step PCR method using primers P1878, P1885, P1887, and P1893 to construct a 3 × FLAG tag at the N terminus of *pog1* and replace the endogenous promoter with the thiamin-repressible *nmt81* promoter via the pFA6a-kanMX6-P81nmt1-3FLAG vector [19]. Unless otherwise indicated, experiments using the *nmt81-pog1* strain were performed in YES medium, which provides sufficient endogenous thiamine to partially repress the *nmt81* promoter while maintaining the low level of Pog1 expression required for cell viability. Complete repression of *nmt81*-driven expression was examined in EMM2 medium supplemented with 10 µg/mL thiamine [19]. Gene deletions and mutations used in this study have been described previously for *swi1*∆ (*swi1*::*natMX6*) [20] and *rad52-12PK*:*KanMX6* [4,21].

The general genetic and molecular biology and biochemical methods for *S. pombe* were performed as previously described [18]. Serial dilution growth assays and drug sensitivity assays were carried out as reported in earlier studies [22,23]. The primer used for strain construction in this study are listed in Table A2.

### 2.2. Cell Culture and RNAi

Human U-2 OS cells and Saos-2 cells were cultured in McCoy’s 5A medium, and TE-11 cells were cultured in RPMI medium (Corning, New York, NY, USA), each supplemented with 10% fetal bovine serum (FBS), 50 U/mL penicillin, and 50 µg/mL streptomycin.

Constitutive short hairpin RNA (shRNA)-mediated gene silencing was performed using the lentiviral vector pLKO.1-PURO encoding scrambled control, Timeless shRNA-#3 (CCGGGCCCACACTAACCATTGCATTCTCGAGAATGCAATGGTTAGTGTGGGCTTTTTTG), and Timeless shRNA-#5 (CCGGCGATACCAGATTGAGGATGATCTCGAGATCATCCTCAATCTGGTATCGTTTTTTG) as described previously [24,25]. Timeless shRNA-#4 (CCGGCGAGAACTATTCCTGGCACTTCTCGAGAAGTGCCAGGAATAGTTCTCGTTTTTTG) and Timeless shRNA-#6 (CCGGGCCGCATCATCAAGAACAATACTCGAGTATTGTTCTTGATGATGCGGCTTTTTTG) were also used.

Transfection of siRNA duplexes were carried out as previously described [24,25], using siRNAs purchased from Sigma-Aldrich, St. Louis, MO, USA; Mission Universal Negative Control #1 (SIC001), Timeless-siRNA-#1 (SASI_Hs01_00010403), and Timeless-siRNA-#2 (SASI_Hs01_00010411) were used.

For the propidium iodide (PI) exclusion assay to measure cell viability, both adherent and floating cells were collected, suspended in 1 × PBS, and incubated with 2.5 µg/mL PI. Samples were then diluted to 1:5 in buffer containing 0.1 M HEPES/NaOH (pH7.4), 1.4 M NaCl, and 25 mM CaCl_2_, and subsequently analyzed using the Guava EasyCyte Mini flow cytometer in the Guava Express Plus program (Guava Technology, Hayward, CA, USA). A WST-8 assay was performed using the Cell Counting Kit-8 (Abcam, Cambridge, UK) according to the manufacturer’s instructions to assess cell fitness. Cells were seeded in 96-well plates and treated with the indicated drugs for 48 h. Absorbance was measured at 450 nm with background subtraction at 620 nm while using wells without cells as controls.

### 2.3. mtDNA Copy Number Assay

The total genomic DNA from exponentially growing *S. pombe* and human cells was isolated using the glass bead preparation method, as described by Burke et al. in 2000 [26], and the Quick-DNA^TM^ Miniprep Plus Kit (Zymo Research, Irvine, CA, USA) according to the manufacturer’s instructions. To measure the relative mtDNA copy number, 2 ng of each DNA sample was subjected to qPCR using the primer sets targeting mitochondrial genes and a single-copy nuclear gene (see below). Reactions were performed with the PowerUp^TM^ SYBR^TM^ Green Master Mix (Applied Biosystems, Foster City, CA, USA) on a Bio-Rad CFX96^TM^ Real-Time System coupled to the C1000^TM^ Thermal Cycler (Bio-Rad Laboratories, Hercules, CA, USA). For *S. pombe*, primer pairs for *atp9* (mtDNA, P1874/P1875) and *spo12* (nuclear, P1876/P1877) were used. For human DNA, primer pairs for *ND1* (mtDNA, P1850/P1851), *ND4* (mtDNA, P1889/P1890), *CO1* (mtDNA, P1891/P1892), and *HGB* (nuclear, P1852/P1853) were used. The relative mtDNA copy number was determined by qPCR following normalization to a single-copy nuclear reference gene. Briefly, the ∆Ct values were obtained by subtracting the median Ct of the nuclear gene from the Ct of the mtDNA gene. The relative mtDNA copy number was then calculated as 2^−∆Ct^ and is presented as mtDNA copy number normalized to the nuclear reference locus. This approach permits the direct comparison of the normalized mtDNA copy number between samples and across different mitochondrial loci. All oligonucleotide primers used for the mtDNA copy number assay are listed in Table A3.

### 2.4. ChIP-Seq of Analysis of Rad52-12PK

ChIP samples from mid-log-phase Y4250 (*rad52-12PK*) and Y4256 (*rad52-12PK swi1*∆) strains were prepared as described [27]. Briefly, exponentially growing cells were crosslinked with 3% paraformaldehyde, and chromatin was sheared into 500 to 700 bp fragments using a Misonix Sonicator 3000 (Qsonica, Newtown, CT, USA). Chromatin-associated proteins were immunoprecipitated using the mouse monoclonal anti-V5/Pk (SV5-Pk1, AbD Serotec, Oxford, UK) together with Protein G-coupled Dynabeads (Life Technologies, Carlsbad, CA, USA). Crosslinks were reversed, and DNA was purified from the immunoprecipitates and also from a no-IP input control. DNA quantity and quality were assessed using Qbit fluorometry (Invitrogen, Carlsbad, CA, USA) and an Agilent Bioanalyzer (Agilent Technologies, Santa Clara, CA, USA). Sequencing libraries were prepared using the Takara ThruPLEX DNA-seq 48D kit (Takara Bio, Shiga, Japan) according to the manufacturer’s instructions and sequenced on an Illumina NextSeq500 (Illumina, San Diego, CA, USA), with a target depth of ~15 million reads at 1 × 75 nt. Sequence reads were aligned against the complete *S. pombe* reference sequence (from PomBase [28]: ASM294v2, GCA_000002945.2), including all three nuclear chromosomes and the mitochondrial genome, using BWA-MEM (v0.7.13-r1126) [29] and sambamba [30]. ChIP enrichment was measured using MACS2 (callpeak-B--SPMR-nomodel-keep-dup 1) with merged duplicates with genotype-specific no input controls acting as the background [31]. Fold enrichment (FE) values, called peaks, and read alignments were visualized with the Integrative Genomics Viewer (IGV, Broad Institute, Cambridge, MA, USA) [32].

### 2.5. RNA-Seq and Analysis

The total RNA from triplicate log-phase cultures of wild-types and *swi1*∆ was isolated using the Master Pure^TM^ Yeast RNA Purification Kit (Epicentre Biotechnologies, Madison, WI, USA) following the instructions provided by the supplier. The Illumina TruSeq stranded Total RNA library kit (Illumina) was used for library prep, and sequenced at 1 × 75 nt on an Illumina NextSeq500 targeting ~15 million reads per sample. Alignments were conducted as above, and bedtools coverage [33] was used to count the number of reads that aligned to each gene for each sample.

The total RNA was isolated from U-2 OS cells expressing scrambled control or Timeless shRNA-#3 in biological triplicates using TRIzol reagent. RNA samples were analyzed by Plasmidsaurus using a 3′ end counting RNA-seq workflow. Libraries were prepared by poly(A) mRNA capture with barcoded oligo-dT primers containing UMIs, followed by second-strand synthesis, tagmentation, and Illumina library amplification with dual indices. Sequencing was performed on an Illumina platform to generate ~10 million deduplicated 3′ end reads per sample. Data outputs included FASTQ files, deduplicated BAM files, quality control metrics, and gene count tables for downstream differential expression analysis.

To assess the relative mRNA expression levels of *rad52*, *cmb1*, *zip1*, and *pog1* in *swi1*∆ cells compared to wild-type cells, gene expression values were normalized using the median-of-ratios method provided by *DESeq2* in Bioconductor [34,35]. Data were visualized as a log10 of the normalized expression using the *ggplot* and *ggrepel* packages in R. Gene Set Enrichment Analysis (GSEA), and leading-edge analyses were conducted using the HALLMARK gene sets from the Molecular Signatures Database (MSigDB) with the clusterProfiler package in R.

### 2.6. Western Blotting and Antibodies

Protein extraction and Western blotting from *S. pombe* and human cells were conducted as described [20,25]. The following antibodies were used in this study: anti-FLAG (M2, Sigma-Aldrich), anti-α-tubulin (B-5-1-2, Sigma-Aldrich), anti-tubulin TAT-1, anti-NRF2 (sc-722, Santa Cruz Biotechnology, Dallas, TX, USA), anti-TFAM (sc-28200, Santa Cruz Biotechnology), and anti-phospho-S6K1 (Thr289; #9206, Cell Signaling Technology, Danvers, MA, USA). The Timeless antibody used in this study was described in our previous work [36].

### 2.7. Mitochondrial Functional Assays

Mitochondrial functional assays were performed as described in our previous studies with minor modifications [37]. Briefly, mitochondrial mass was assessed by incubating cells with 100 nM MitoTracker^TM^ Green FM (Invitrogen, Carlsbad, CA, USA). Mitochondrial ROS levels were measured by incubating cells with 5 μM of MitoSox^TM^ Red Mitochondrial Superoxide Indicator (Invitrogen). Mitochondrial membrane potential was determined using 1 µM tetramethylrhodamine ethyl ester perchlorate (TMRE; Invitrogen). For all three assays, adherent cells were washed with PBS to remove the culture medium and detached cells, incubated with the respective reagents at 37 °C for 30 min, harvested in 2.5% trypsin–EDTA, and resuspended in 400 μL growth medium. Samples were immediately analyzed on the Guava EasyCyte Mini flow cytometer using the Guava Express Plus program (Guava Technologies, Hayward, CA, USA). Debris and dead cells were excluded by flow cytometric gating prior to quantification of fluorescence intensity.

### 2.8. Immunofluorescence Microscopy

Immunofluorescence microscopy of human cells was performed as described [25]. Briefly, U-2 OS cells were seeded onto chambered polystyrene slides (Corning/Falcon); fixed with 4% formaldehyde in PBS; permeabilized with 100 mM Tris-HCl (pH 7.4), 50 mM EDTA, and 0.5% Triton X-100; and blocked with 5% FBS in PBS. Cells were then incubated with an anti-NRF2 antibody (C-20, sc-722), followed by an Alexa Fluor 568-conjugated anti-rabbit IgG secondary antibody. DNA was counterstained using ProLong Gold Antifade Mountant with DAPI (Thermo Fisher Scientific, Waltham, MA, USA). Images were captured using an EVOS FL Auto Imaging System (Life Technologies) and analyzed with ImageJ 1.52a software (National Institutes of Health, Bethesda, MD, USA).

## 3. Results

### 3.1. Swi1 Deletion Impairs Mitochondrial Genome Integrity in S. pombe

**Figure 1:**
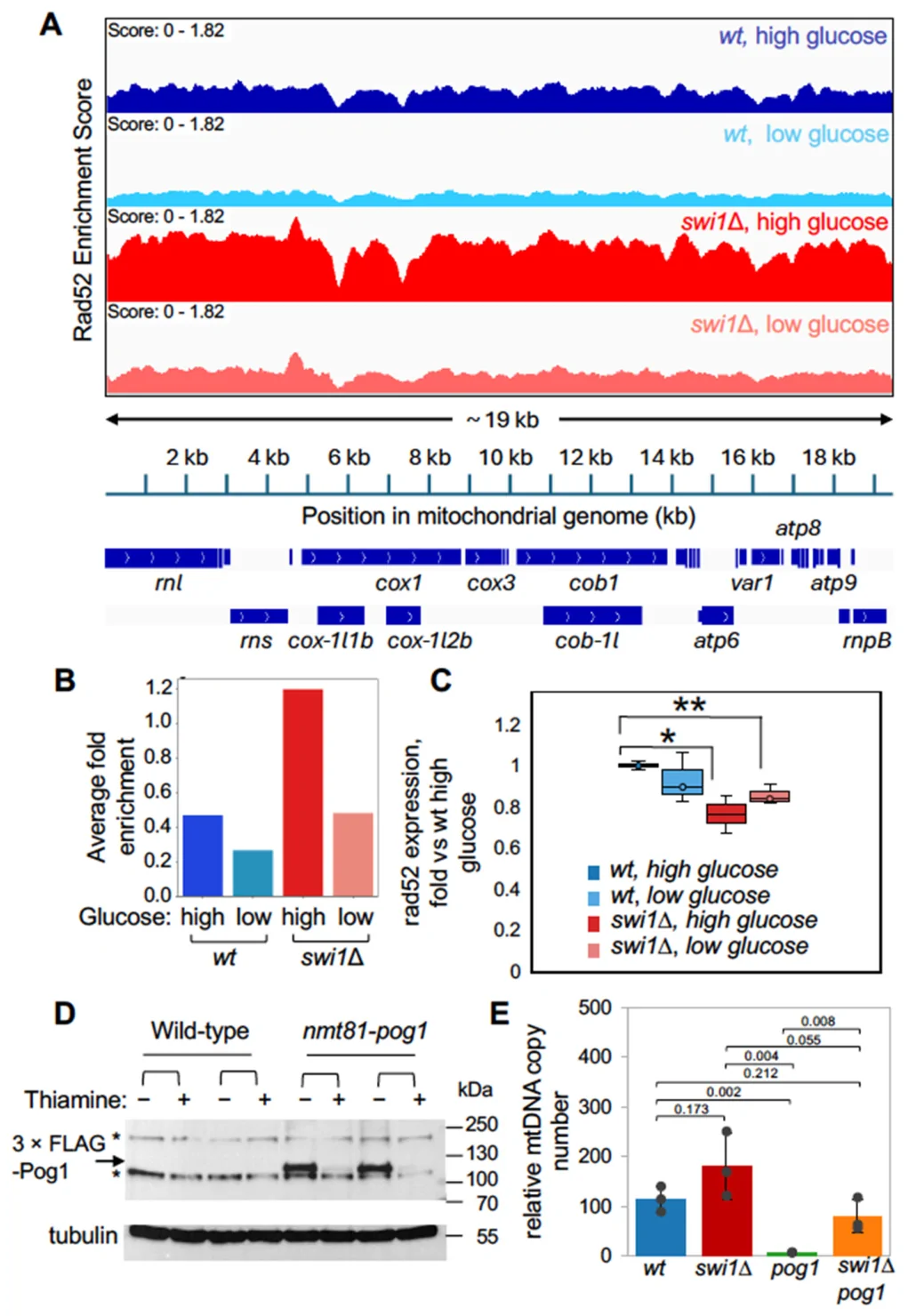
The *swi1* deletion impairs mitochondrial genome integrity in *S. pombe.* (**A**) ChIP-seq analysis of Rad52 distribution across mitochondrial DNA (mtDNA) in asynchronous cultures of wild-type (blue) and *swi1*∆ (red) *S. pombe* cells grown in YES medium containing 5% (dark shading) and 0.1% (light shading) glucose. Average Rad52 enrichment from two-independent cultures is shown as fold enrichment scores (y-axis). Mitochondrial genome coordinates (x-axis; in kilobases, kb) were downloaded from PomBase (www.pombase.org) [28]. (**B**) Average Rad52 enrichment scores across the mitochondrial genome from two independent ChIP-seq experiments are displayed as a bar graph. (**C**) RNA-seq analysis of *rad52* transcript abundance in wild-type and *swi1*Δ *S. pombe* cells grown under glucose-rich (5%) and glucose-limited (0.1%) conditions. The *rad52* transcript abundance was determined from normalized RNA-seq counts. Bars represent mean ± SD. *p*-values were determined by one-tailed Student’s *t*-test. * *p* < 0.05 and ** *p* < 0.01. (**D**) Wild-type cells and cells engineered to express 3 × FLAG-Pog1 under the control of the *nmt81* promoter (*nmt81-pog1*) were grown in EMM2, either in the presence or absence of 10 µg/mL thiamine (B1), and subjected to Western blotting analysis to detect 3 × FLAG-Pog1. Tubulin was used as a loading control. Asterisks indicate non-specific bands. (**E**) Genomic DNA was isolated from *S. pombe* cells with the indicated genotypes and used to determine the relative mtDNA copy number by qPCR. The relative mtDNA copy number was determined using the mitochondrial *atp9* locus and normalized to the single-copy nuclear gene *spo12*. *n* = 3. Bars represent mean ± SEM. *p*-values were determined by two-tailed Student’s *t*-test.

Because Swi1 deficiency causes replication stress and accumulation of DNA damage in the nuclear genome in *S. pombe*, resulting in increased Rad52 association at several genomic loci, including telomeres, rDNA, and ectopically introduced *LacO* repeats [4], we investigated whether loss of Swi1 also affects mitochondrial genome stability. To address this question, we performed ChIP-seq analysis of Rad52 association across the *S. pombe* mitochondrial genome under glucose-limited and glucose-rich conditions (Figure 1A). Sequence reads were aligned to the complete *S. pombe* reference genome including both the nuclear and mitochondrial chromosomes (see Section 2).

In wild-type cells, Rad52 association showed only a modest increase in glucose-rich medium (high glucose) relative to glucose-limited conditions (low glucose). In contrast, *swi1*Δ cells (lacking the *S. pombe* Timeless ortholog) displayed markedly elevated Rad52 association broadly across the mitochondrial genome (Figure 1A,B). Notably, this elevated Rad52 association in *swi1*∆ cells persisted even under glucose-limited conditions, in which wild-type cells showed lower levels of Rad52 association (Figure 1A,B). Because the increased Rad52 signal was distributed throughout the mitochondrial genome rather than confined to discrete loci, these findings suggest a genome-wide increase in the association of Rad52 with mitochondrial DNA sequences in the absence of Swi1. Although previously reported nuclear mitochondrial DNA segments (NUMTs) in *S. pombe* cannot be completely excluded as a minor source of mapped reads, they represent only a limited and fragmented subset of the mitochondrial genome [38,39] and are therefore unlikely to account for the broad distribution of Rad52 association observed across the mitochondrial genome.

Importantly, RNA-seq analysis did not reveal an increased *rad52* transcript abundance in *swi1*∆ cells. Instead, *rad52* transcript levels in *swi1*∆ cells were modestly reduced under glucose-rich conditions and were not significantly different from those in wild-type cells under glucose-limited conditions (Figure 1C). Likewise, glucose availability did not significantly alter *rad52* transcript levels within either the wild-type or *swi1*∆ backgrounds (Figure 1C). These findings indicate that increased *rad52* transcription is unlikely to account for the increased ChIP-seq signal. Furthermore, these data reflect increased association of Rad52 with mitochondrial DNA and do not directly assess total Rad52 protein abundance or mitochondrial localization. Together, these results are consistent with increased mitochondrial genome stress or instability in *swi1*∆ cells.

**Figure 2:**
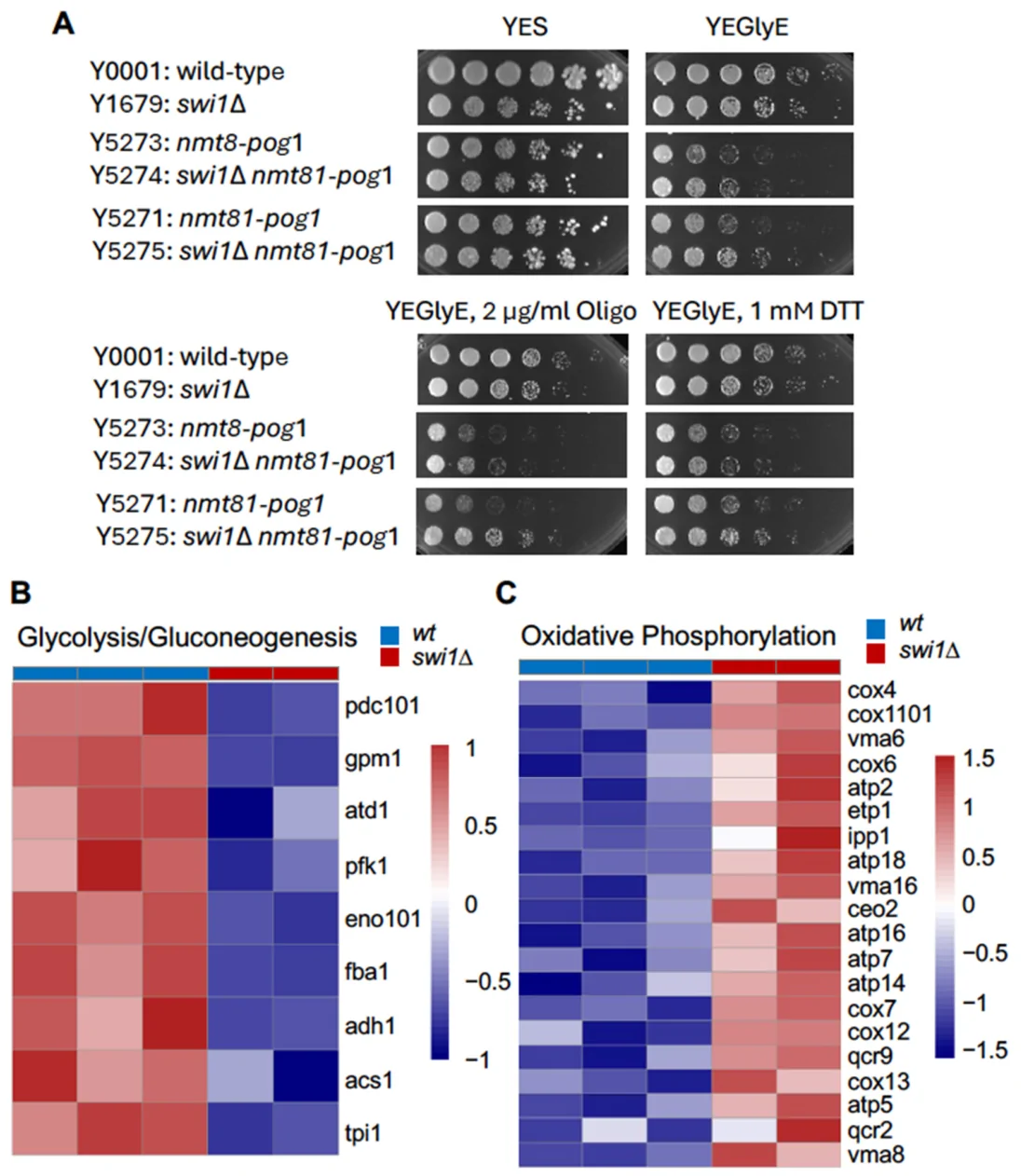
The *swi1* deletion partially rescues growth defects of *pog1*-deficient cells under respiratory conditions. (**A**) *S. pombe* cells with the indicated genotypes were grown to mid-log phase in YES medium, and fivefold serial dilutions were spotted onto YES agar or YEGlyE agar media supplemented with 2 µg/mL of oligomycin or 1 mM DTT. Representative images of repeat experiments are shown. The indicated strains were analyzed on the same YES and YEGlyE agar plates; the images have been cropped to show only the strains relevant to this study. (**B**,**C**) RNA-seq datasets were subjected to KEGG pathway analysis and visualized as a heatmap of differentially expressed genes (DEGs). Expression data are represented as z-scores per each gene, with red representing high expression and blue indicating low expression.

In *S. pombe*, mtDNA is replicated by DNA polymerase γ, encoded by the *pog1^+^* gene. The deletion of *pog1* leads to a loss of mtDNA and results in a petite phenotype characterized by severe growth defects [40]. To conditionally reduce *pog1* expression, we replaced its native promoter with the thiamine-repressible *nmt81* promoter at the endogenous locus and expressed Pog1 as a 3 × FLAG fusion protein. To confirm repression of the *nmt81-pog1* allele, cells were cultured in EMM2 in the presence or absence of 10 µg/mL thiamine. As expected, thiamine treatment markedly reduced 3 × FLAG-Pog1 protein levels (Figure 1D). Subsequent phenotypic analyses of the *nmt81-pog1* strain were performed in YES medium, which partially represses *nmt81*-driven expression while maintaining sufficient Pog1 for cell viability. Although *nmt81-pog1* cells showed mild growth defects, they were still viable (Figure 2A), allowing for their characterization.

To examine the effect of Swi1 loss on mitochondrial genome maintenance, we quantified the mitochondrial DNA (mtDNA) copy number by comparing the mitochondrial gene *atp9*^+^ with the single-copy nuclear gene *spo12*^+^. As a control, *nmt81-pog1* cells displayed a pronounced reduction in the mtDNA copy number (Figure 1E), consistent with the established role of Pog1 in mtDNA replication. In contrast, *swi1*∆ cells showed a modest increase in the mtDNA copy number despite exhibiting an increased Rad52 association with mitochondrial genome (Figure 1E). This paradoxical increase suggests that the loss of Swi1 activates a compensatory mtDNA amplification response, potentially offsetting replication stress or damage within the mitochondrial genome.

### 3.2. Swi1 Deletion Alleviates Growth Defects Caused by Mitochondrial DNA Polymerase Depletion in S. pombe

To examine the effect of Swi1 and Pog1 deficiency on cell growth, we performed serial-dilution growth assays on both glucose- and glycerol-containing solid media (Figure 2A). While glucose allows cells to generate ATP via glycolysis, glycerol forces reliance on mitochondrial respiration. In the glucose medium, *nmt81-pog1* cells exhibited modest growth delays compared with wild-types (Figure 2A). As expected, *nmt81-pog1* cells showed severe growth defects on the glycerol medium. This growth inhibition was further exacerbated in the presence of oligomycin or dithiothreitol (DTT), both of which negatively impact mitochondrial fitness (Figure 2A), confirming the critical role of Pog1 in mitochondrial respiration. The *swi1*∆ cells had modest growth defects in the glucose-containing medium, consistent with the well-known role of Swi1 in preventing replication stress. Strikingly, when cultured on the glycerol medium, *swi1*∆ cells grew comparably to wild-type cells (Figure 2A).

We then constructed *swi1*∆ *nmt81-pog1* double-mutant cells and compared their growth with wild-type and single-mutant strains. Interestingly, *swi1*∆ *nmt81-pog1* cells displayed a modest but reproducible improvement in growth relative to *nmt81-pog1* single mutants in the glycerol medium (Figure 2A). Similar growth improvements were also observed in independent isolates with the same genotypes (Figure 2A). This growth enhancement was particularly evident when cells were cultured in the presence of oligomycin (Figure 2A), which inhibits ATP synthesis.

To determine whether this partial growth recovery correlates with changes in mitochondrial DNA contents, we quantified the mtDNA copy number in double-mutant cells. The mtDNA copy number was significantly increased in *swi1*∆ *nmt81-pog1* cells compared to *nmt81-pog1* cells (Figure 1E), suggesting a positive correlation between the mtDNA copy number and respiratory growth capacity. Notably, *swi1*∆ cells also exhibit an increased mtDNA copy number (Figure 1E), which may contribute to their ability to maintain growth under respiratory conditions.

To examine the effect of Swi1 deletion and subsequent mtDNA enrichment on broader mitochondrial function, we analyzed RNA-seq datasets using KEGG pathway enrichment analysis. Pathway-level analysis revealed enrichment of transcriptional changes consistent with reduced glycolytic activity (Figure 2B; Supplementary Figure S1) and upregulated oxidative phosphorylation (Figure 2C; Supplementary Figure S2) in *swi1*∆ cells, suggesting metabolic reprograming in response to loss of Swi1. These pathway-level trends are consistent with the improved growth of *swi1*∆ cells on glycerol medium (Figure 2A) and support the possibility of increased reliance on mitochondrial respiration.

### 3.3. Timeless Depletion Results in Loss of Mitochondrial Fitness in Human U-2 OS Cells

**Figure 3:**
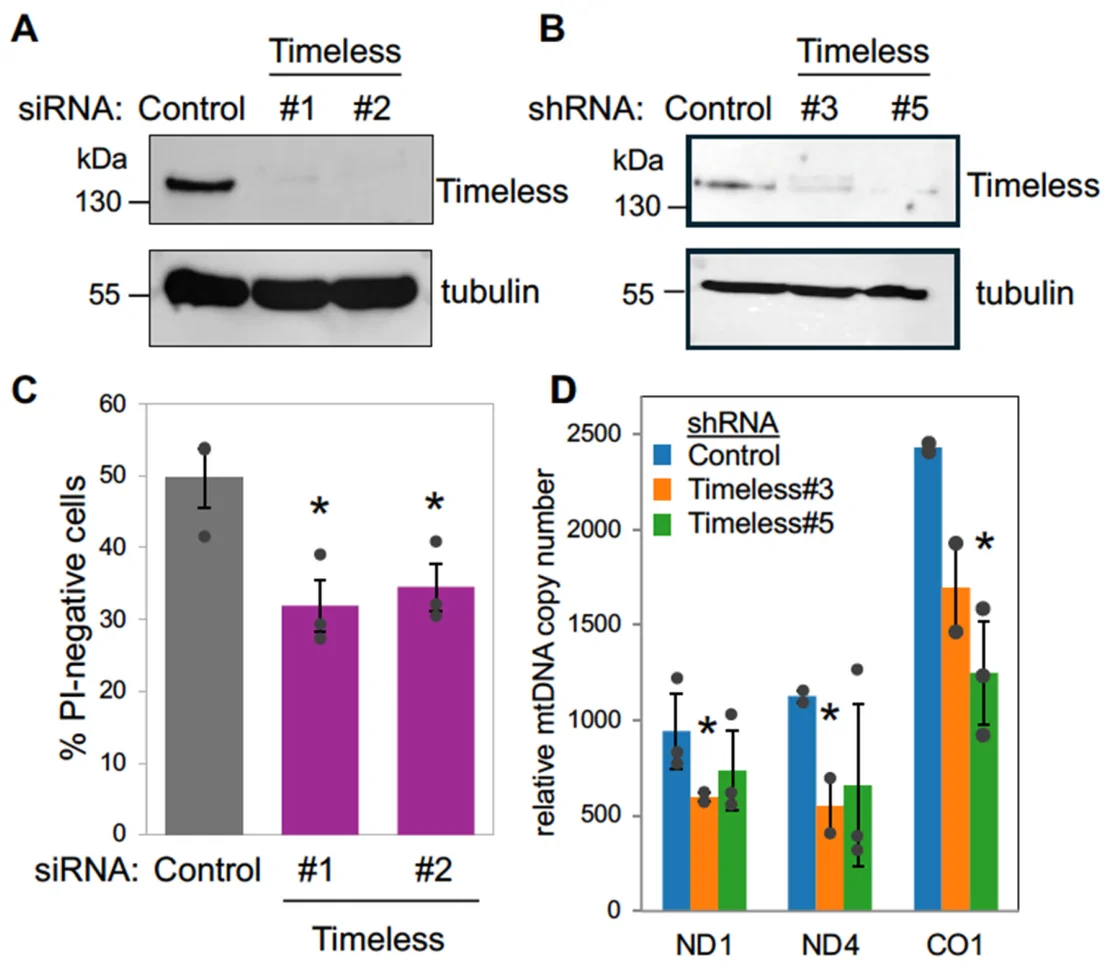
Timeless depletion results in a reduced mtDNA copy number in U-2 OS cells. (**A**) U-2 OS cells were transfected with the indicated siRNAs and subjected to Western blot analysis to detect the Timeless protein. Tubulin was used as a loading control. (**B**) U-2 OS cells stably expressing the indicated shRNAs were processed for Western blot analysis to detect Timeless and tubulin. (**C**) Cell viability was assessed two days after transient siRNA transfection in U-2 OS cells using propidium iodide (PI) exclusion. (**D**) Genomic DNA was isolated from adherent U-2 OS cells stably expressing the indicated shRNAs after removing the culture medium and PBS washing, and was used to determine the mtDNA copy number by qPCR. The relative mtDNA copy number was determined using the mitochondrial loci *ND1*, *ND4*, and *CO1* and normalized to the single-copy nuclear gene *HGB*. *n* = 3. Bars represent mean ± SEM. * *p* < 0.05.

To examine the role of Timeless/Swi1 in mitochondrial maintenance in higher eukaryotes, we depleted Timeless in a human U-2 OS osteosarcoma cell line using siRNA or shRNA-expressing lentiviral vectors (Figure 3A,B). Timeless-depleted cells showed decreased viability (Figure 3C), although they were able to grow and divide continuously.

The mtDNA copy number was quantified by comparing the mitochondrial gene *ND1* with the single-copy nuclear gene *HGB*. In striking contrast with the *S. pombe* results, Timeless depletion led to a reduction in the mtDNA copy number of U-2 OS cells (Figure 3D). Similar decreases were observed when ND4 and CO1 genes were used as mitochondrial references (Figure 3D). Although the difference could reflect a cell-cycle delay, where Timeless-depleted cells spend more time in S and G2 phases due to slowed replication, our findings indicate that, unlike in *S. pombe*, Timeless loss in U-2 OS cells compromises mitochondrial genome maintenance rather than triggering a compensatory increase in the mtDNA copy number. Thus, DNA damage resulting from Timeless depletion is associated with reduced genomic instability of mitochondrial DNA.

**Figure 4:**
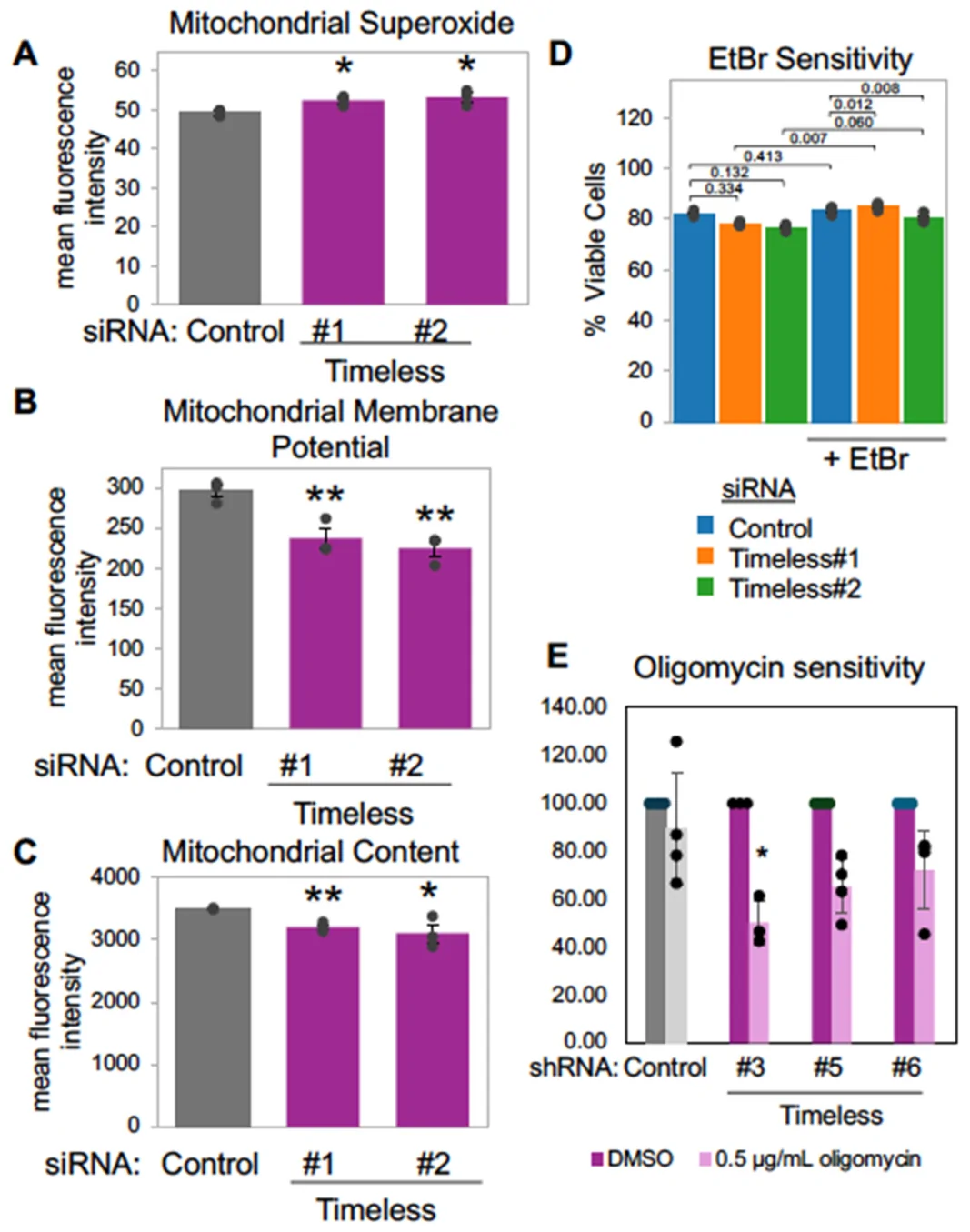
Timeless depletion impairs mitochondrial functions in U-2 OS cells. (**A**–**C**) U-2 OS cells were transiently transfected with the indicated control of Timeless siRNAs, and mitochondrial superoxides (**A**), membrane potential (**B**), and mitochondrial content (**C**) were measured as described in the Section 2. (**D**) U-2 OS cells transfected with the indicated siRNAs were cultured in the presence of 50 ng/mL ethidium bromide (EtBr) for 5 days. Cell viability was determined using Trypan Blue exclusion. (**E**) U-2 OS cells transfected with the indicated siRNAs were cultured in the presence of 0.5 µg/mL oligomycin for 2 days, and cell fitness was measured using a WST-8 assay. *n* = 3. Bars represent mean ± SEM. * *p* < 0.05 and ** *p* < 0.01.

To evaluate mitochondrial function in U-2 OS cells, we measured mitochondrial reactive oxygen species (mtROS) using siRNAs targeting Timeless (Figure 3A). Timeless depletion modestly but reproducibly increased mtROS levels (Figure 4A; Supplementary Figure S6). We then assessed mitochondrial membrane potential in live cells using TMRE staining and found that siRNA-mediated Timeless knockdown caused a statistically significant reduction in membrane potential (Figure 4B; Supplementary Figure S7). The combination of increased mtROS and decreased TMRE signals was indicative of mitochondrial dysfunction, suggesting that the loss of Timeless negatively affected the maintenance of mitochondrial integrity in U-2 OS cells.

To further assess mitochondrial content, we stained cells with MitoTracker. Timeless-depleted U-2 OS cells showed a statistically significant decrease in MitoTracker signal (Figure 4C; Supplementary Figure S8), consistent with reduced mitochondrial mass and decreased mitochondrial membrane potential. We next examined cell viability following siRNA-mediated Timeless depletion in U-2 OS cells combined with low-dose ethidium bromide treatment, which inhibits DNA polymerase γ activity without affecting nuclear DNA [41,42]. The trypan blue exclusion assay revealed a modest but significant increase in cell death when Timeless depletion was combined with Pol γ inhibition (Figure 4D), indicating that the loss of Timeless sensitizes cells to Pol γ inhibition. We also assessed cell fitness in U-2 OS cells following oligomycin treatment. Although the effect was modest, Timeless depletion reduced cell fitness under these conditions (Figure 4E).

Together, these observations of decreased mtDNA content, elevated mtROS, reduced TMRE staining, reduced MitoTracker signaling, and increased EtBr and oligomycin sensitivity suggest that Timeless deletion impairs mitochondrial genome stability and compromises mitochondrial fitness in U-2 OS cells.

### 3.4. Context-Dependent Effects of Timeless Depletion on Mitochondrial Homeostasis in Human Cells

**Figure 5:**
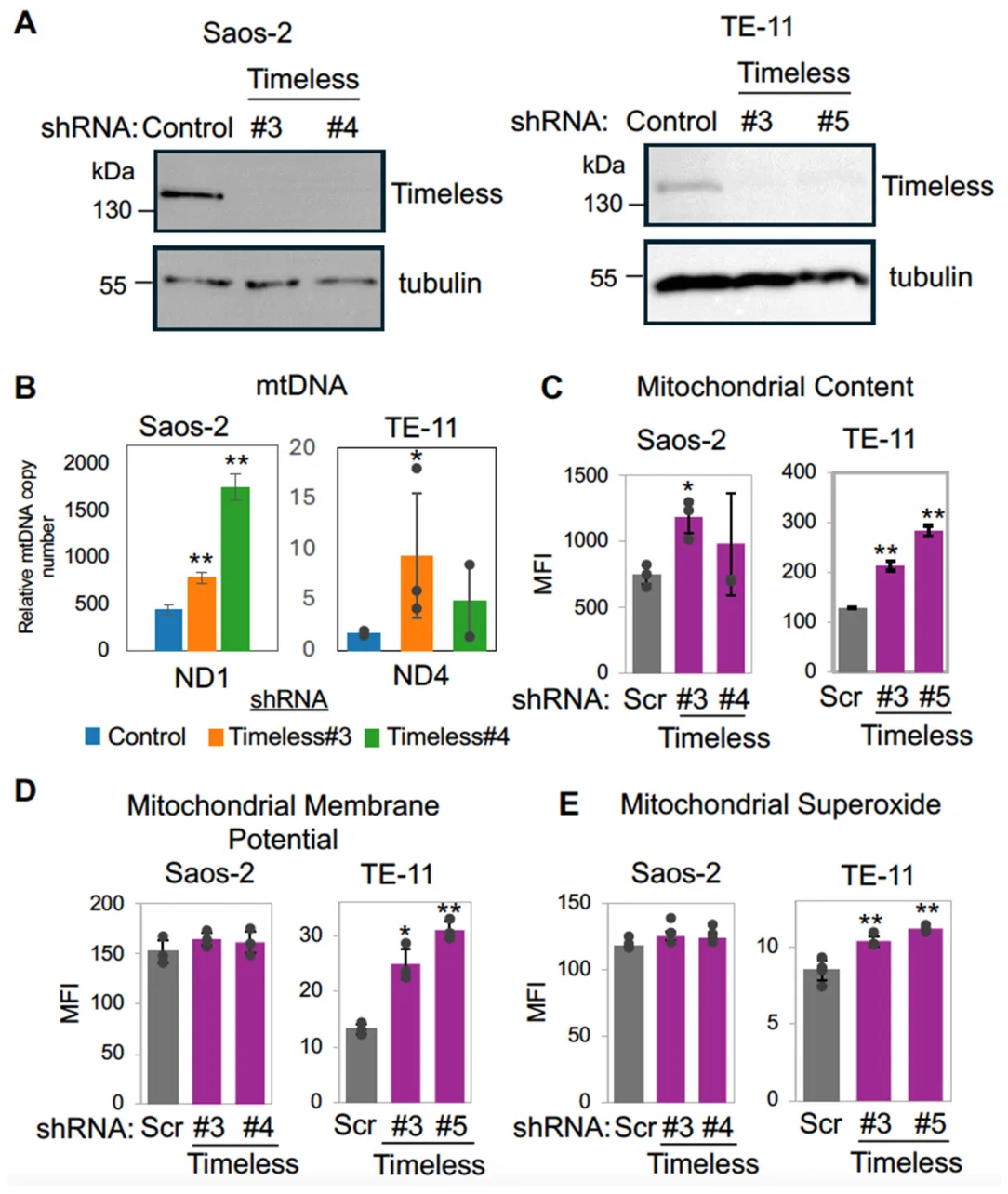
Timeless depletion alters mitochondrial regulation in TE-11 and Saos-2 cells. (**A**) Cell lysates were prepared from Saos-2 and TE-11 cells expressing the indicated shRNAs and probed for Timeless protein by Western blotting. Tubulin protein was used as a loading control. (**B**) Genomic DNA isolated from the indicated shRNA-expressing cells was subjected to qPCR analysis to determine the relative mtDNA copy number using the mitochondrial loci *ND1* and *ND4*. Values were normalized to the single-copy nuclear gene *HGB*. (**C**–**E**) Determination of mitochondrial content (**C**), membrane potential (**D**), and superoxides (**E**) were measured in the indicated cells as described in the Section 2. *n* = 3. Bars represent mean ± STDEV. * *p* < 0.05 and ** *p* < 0.01. MFI: mean fluorescence intensity.

To further investigate whether the mitochondrial phenotypes associated with Timeless depletion were cell-type specific or more broadly conserved, we extended our analysis to additional human cell lines, including Saos-2 and TE-11 cells (Figure 5A). Although both U-2 OS and Saos-2 cells are derived from osteosarcoma, Timeless depletion in Saos-2 cells, in contrast to U-2 OS cells, resulted in an increase in the mtDNA copy number, consistent with observations in *S. pombe* (Figure 5B). Similarly, Timeless depletion in TE-11 esophageal squamous cell carcinoma cells led to an elevated mtDNA copy number (Figure 5B). These increases were accompanied by enhanced MitoTracker signals, suggesting increased mitochondrial mass (Figure 5C; Supplementary Figures S9 and S12).

Consistent with the findings from *S. pombe*, the elevated mtDNA copy number in Timeless-depleted TE-11 cells correlated with increased mitochondrial membrane potential, as measured by TMRE staining (Figure 5D; Supplementary Figure S13). In contrast to U-2 OS cells, in which TMRE signals decreased upon Timeless depletion, Saos-2 cells did not exhibit a reduction in TMRE signals (Figure 5D; Supplementary Figure S10).

While Timeless depletion produced divergent effects on the mtDNA copy number, MitoTracker signals, and TMRE across U-2 OS and TE-11 cells, both cell lines exhibited increased mitochondrial ROS, as measured by MitoSOX (Figure 5E; Supplementary Figures S11 and S14). This consistent elevation in mtROS suggests that Timeless depletion induces a mitochondrial stress response and perturbs mitochondrial homeostasis across these cell lines.

Collectively, these results indicate that Timeless contributes to the regulation of mitochondrial homeostasis in human cells; however, the phenotypic outcomes are cell-type dependent and likely reflect the underlying differences in cellular context.

### 3.5. Timeless Depletion Activates Antioxidant Stress Pathways

**Figure 6:**
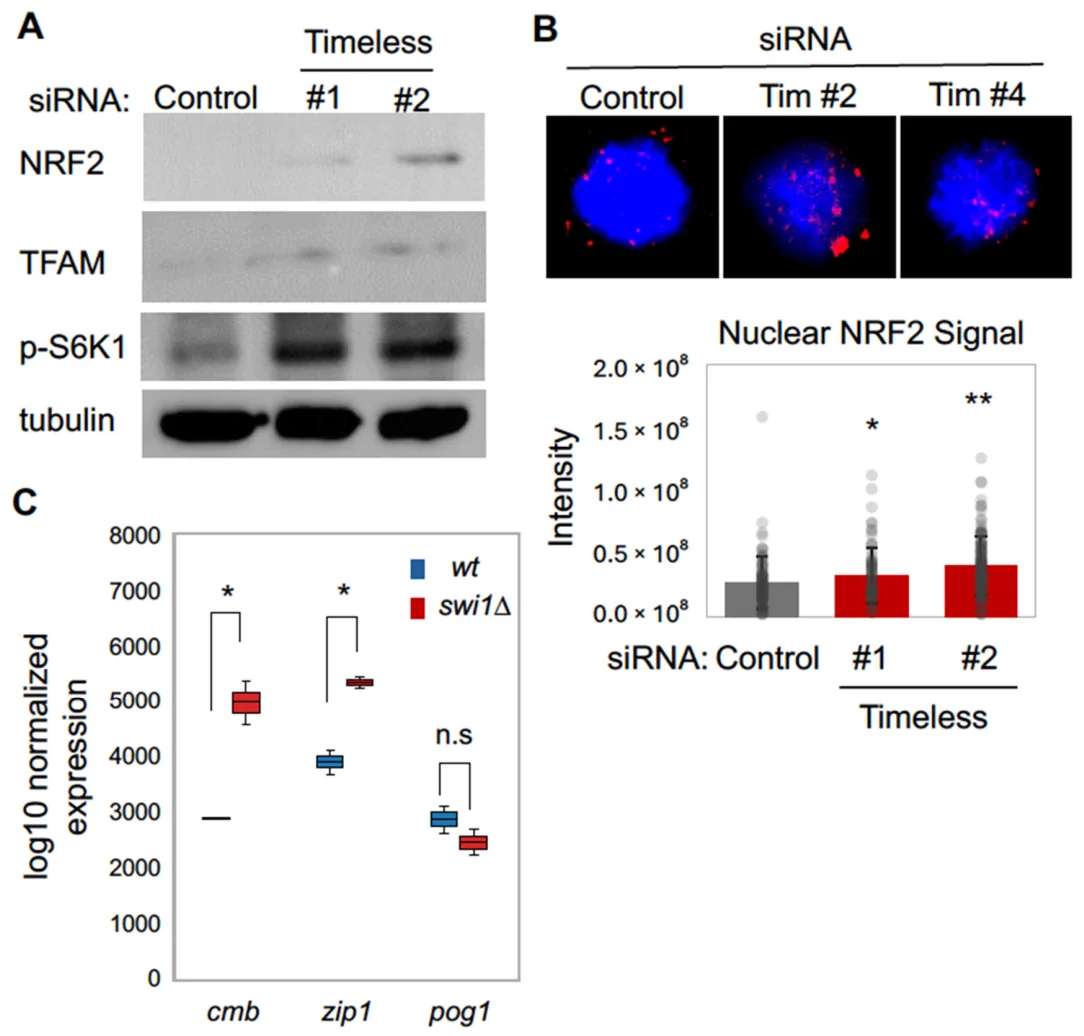
Oxidative stress response activation upon Timeless/Swi1 loss. (**A**) Cell lysates were prepared from U-2 OS cells transfected with the indicated siRNAs and probed for NRF2, TFAM, and phospho-S6K1 by Western blotting. Tubulin was used as a loading control. (**B**) Immunofluorescence was performed in U-2 OS cells to determine the nuclear localization of NRF2 relative to control cells. Data are represented as the mean corrected intensity of the nuclear signal. At least 50 cells were counted for each replicate. *n* = 2. Bars represent mean ± STDEV. *p*-values were determined by Mann–Whitney U tests. * *p* < 0.05 and ** *p* < 0.01. (**C**) Box plots for RNA-seq analysis of gene expression in *S. pombe* was determined for *cmb*, *zip1*, and *pog1* in wild-type (blue) and *swi1*∆ (red) cells. Expression values are represented as log10 of the normalized expression values. The boxes were limited by the 25th and 75th percentiles. The horizontal black lines represent the median value. The whiskers extend to the largest and smallest data points that are no more than 1.5 times the interquartile range from the box. *p*-values were determined by student *t*-test, * *p* < 0.05.

mtROS is known to act as a signaling cue to activate the mTOR pathway. Indeed, in U-2 OS cells, Timeless depletion resulted in elevated phosphorylation of S6K1 (Figure 6A), a downstream target of mTOR. mtROS also triggers the oxidative stress response. Consistently, Timeless depletion led to increased levels of NRF2 (NFE2L2) (Figure 6A), a bZIP (basic leucin zipper)-type transcription factor and the master regulator of the oxidative stress response. As a response to cellular stress, NRF2 translocates from the mitochondrion to the nucleus to trigger transcription of antioxidant proteins [43]. Timeless depletion in U-2 OS cells led to an increase in nuclear NRF2 signals (Figure 6B), suggesting that Timeless depletion activates antioxidant defense pathways. Furthermore, we observed upregulation of TFAM, a nuclear-encoded transcription factor essential for mitochondrial DNA transcription, replication, and packaging (Figure 6A). This increase likely reflects a compensatory effort to stabilize and maintain mitochondrial DNA in response to dysfunction.

To assess whether similar responses are activated in *S. pombe*, we analyzed *S. pombe* RNA-seq data. In *S. pombe*, the *zip1*^+^ gene encodes a bZIP transcription factor Zip1, which is functionally related to human NRF2 [44,45]. The expression of *zip1* was significantly elevated with the absence of Swi1 (*swi1*∆ cells), while *pog1* mRNA levels were unchanged (Figure 6C). Furthermore, transcription of the *S. pombe* TFAM ortholog *cmb1*^+^ was also elevated in *swi1*∆ cells (Figure 6C), suggesting that, although mitochondrial fitness phenotypes differ from those in human cells, Swi1 deficiency activates mitochondrial transcription and stress response pathways.

### 3.6. Transcriptomic Analyses Reveal Conserved Metabolic Reprogramming Toward Oxidative Metabolism upon Timeless/Swi1 Loss

Building on the observed mitochondrial phenotypes and activation of oxidative stress responses, we next sought to determine whether Timeless depletion is associated with broader metabolic alterations. For this purpose, we analyzed RNA-seq datasets using Gene Set Enrichment Analysis (GSEA), Gene Ontology (GO), and KEGG pathway enrichment analyses.

**Figure 7:**
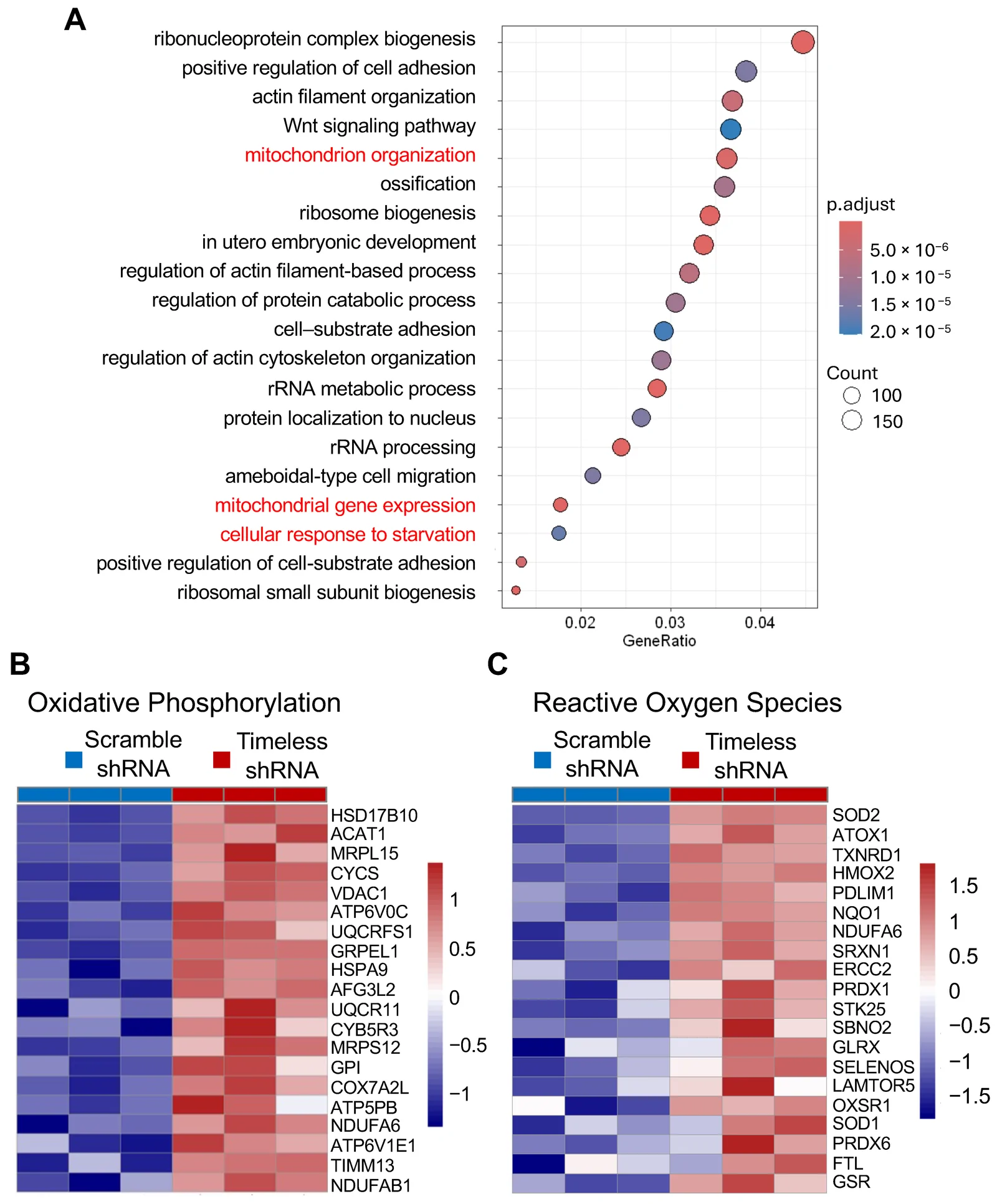
Transcriptome-wide mitochondrial stress response to Timeless/Swi1 loss. (**A**–**C**) RNA-seq datasets were subjected to GO and GSEA pathway analyses. (**A**) GO results were visualized as a dot plot, with the size of dots representing the number of DEGs in the annotated gene set and the color indicating the *p*-value. (**B**,**C**) GSEA analyses were conducted using the HALLMARK gene sets and visualized as a heatmap of DEGs. Data were normalized by obtaining z-scores for each gene, with red representing high expression and blue indicating low expression.

In U-2 OS, Timeless depletion was associated with significant dysregulation of gene sets related to mitochondrial gene expression and mitochondrion organization, as identified by KEGG analysis (Figure 7A). GSEA and leading-edge analysis further revealed enrichment of oxidative phosphorylation pathways following Timeless depletion (Figure 7B; Supplementary Figures S3 and S5). These pathway-level transcriptional changes are consistent with our observations in *S. pombe* (Figure 2; Supplementary Figure S2) and with functional phenotypes, in which *swi1*∆ cells exhibit an elevated mitochondrial DNA copy number (Figure 1E) and improved growth under respiratory conditions (Figure 2A). Together, these findings are consistent with metabolic reprogramming characterized by reduced glycolytic activity and enhanced reliance on oxidative metabolism following Timeless/Swi1 deficiency. The concurrent enrichment of pathways related to mitochondrial organization further suggests that this metabolic shift may be accompanied by broader changes in mitochondrial homeostasis rather than a uniform enhancement of mitochondrial function.

Notably, pathways associated with oxidative stress responses were also enriched following Timeless depletion. These included elevated expressions of SOD1 and SOD2 (Figure 7C; Supplementary Figure S4), consistent with increased mitochondrial superoxides and a compensatory antioxidant response. These transcriptional changes are consistent with our functional data demonstrating increased mitochondrial ROS (Figure 4A and Figure 5E) and activation of NRF2-dependent stress pathways (Figure 6A,B).

Collectively, these results suggest that a Timeless/Swi1 loss is associated with conserved metabolic and stress response reprogramming across species. The transcriptomic analyses support a model in which Timeless/Swi1 deficiency is accompanied by alterations in mitochondrial function, oxidative metabolism, and cellular redox homeostasis.

## 4. Discussion

Our findings reveal a previously unappreciated connection between Timeless (Swi1 in *S. pombe*), mitochondrial genome maintenance, and overall mitochondrial fitness. Although Timeless is best known for its nuclear functions in replication fork protection and genome stability [1,2,3], our data suggest that the loss of Timeless/Swi1 is associated with significant alterations in mitochondrial homeostasis, particularly under conditions of replication stress or impaired mitochondrial function.

In *S. pombe*, the loss of Swi1 resulted in a marked enrichment of Rad52 association with mitochondrial DNA sequences across the mitochondrial genome (Figure 1A), suggesting altered mitochondrial genome homeostasis. One limitation of this analysis is that the compact organization of the *S. pombe* mitochondrial genome, including the extensive overlap among transcriptional units, makes it difficult to define promoter, gene body, and terminator regions with the same confidence as in the nuclear genome. Furthermore, the increase in Rad52 association was broadly distributed across the mitochondrial genome rather than concentrated at discrete loci, limiting meaningful assessment of feature-specific recruitment patterns. In addition, because *swi1*Δ cells exhibit an increased mtDNA copy number, differences in mitochondrial DNA abundance may contribute, in part, to the mitochondrial ChIP-seq signal. Furthermore, the ChIP-seq data do not directly demonstrate mitochondrial localization of Rad52. Therefore, these findings should be interpreted as demonstrating increased association of Rad52 with mitochondrial DNA sequences rather than as evidence of mitochondrial localization of Rad52 or as a quantitative comparison of Rad52 occupancy between the nuclear and mitochondrial genomes.

Unexpectedly, *swi1*Δ cells exhibited an increase in mtDNA copy number (Figure 1D), which correlated with improved growth under respiratory conditions. The estimated mtDNA copy number in wild-type cells (~100–200 copies per nuclear genome) is consistent with previously reported values for actively growing *S. pombe* cells [40], supporting the biological relevance of our qPCR-based measurements. Moreover, the deletion of *swi1* partially alleviated the growth defects and mtDNA loss associated with reduced expression of DNA Polymerase γ (Pog1) (Figure 2A), suggesting that Swi1 deficiency activates compensatory mechanisms that promote mitochondrial genome amplification and sustain respiration despite replication stress. One possible contributing factor to the increased mtDNA copy number is the replication stress-induced cell-cycle delay and increased cell size previously reported in *swi1*Δ cells [46,47]. Because the mtDNA copy number has been reported to scale with cell size and continues increasing during cell-cycle arrest in budding yeast [48], these physiological changes may contribute, at least in part, to the elevated mtDNA copy number observed in *swi1*Δ cells. However, importantly, the increased mtDNA copy number was accompanied by improved respiratory growth, enhanced resistance to respiratory stress, and transcriptional reprogramming consistent with increased oxidative phosphorylation (Figure 2). These observations suggest that altered cell-cycle progression and increased cell size are unlikely to fully account for the mitochondrial phenotypes associated with Swi1 deficiency.

Consistent with these functional observations, RNA-seq analyses coupled with KEGG pathway enrichment revealed that *swi1* deletion is associated with broad metabolic reprogramming. In particular, pathway-level analyses revealed transcriptional changes consistent with reduced glycolytic activity and enhanced oxidative phosphorylation (Figure 2B,C; Supplementary Figures S1 and S2). These transcriptomic changes support a shift away from glycolytic metabolism toward increased reliance on mitochondrial respiration. Together with the increased mtDNA copy number and improved growth in glycerol-based respiratory conditions (Figure 1E and Figure 2A), these findings indicate that Swi1 deficiency promotes a respiration-adapted metabolic state.

While human TE-11 and Saos-2 cells displayed mitochondrial phenotypes similar to *S. pombe* (Figure 5), Timeless depletion in U-2 OS cells led to a reduced mtDNA copy number and mitochondrial mass, elevated mitochondrial ROS, and decreased mitochondrial membrane potential, and increased sensitivity to Pol γ inhibition (Figure 3 and Figure 4). Although the flow cytometric measurements of mitochondrial ROS, membrane potential, and mitochondrial content provide indirect assessments of mitochondrial physiology and may be influenced by multiple biological and technical factors, the observed changes were supported by complementary analyses, including mtDNA copy number measurements, sensitivity to mitochondrial stressors, and transcriptomic profiling. Although mitochondrial responses varied across cell lines, these findings suggest that Timeless depletion is associated with altered mitochondrial homeostasis in human cells. The divergent phenotypes observed in *S. pombe* and human cells further suggest context-dependent responses to the loss of Timeless/Swi1. Nevertheless, both systems exhibited upregulation of TFAM (Cmb1 in *S. pombe*) and the bZIP-type transcription factor NRF2 (Zip1 in *S. pombe*), consistent with activation of mitochondrial transcriptional and antioxidant stress responses as compensatory mechanisms.

Importantly, RNA-seq analyses in U-2 OS cells further revealed enrichment of oxidative phosphorylation and reactive oxidative species signatures, as determined by GSEA, GO and KEGG analyses (Figure 7). Consistent with this, the pathways associated with oxidative stress responses were enriched, and the transcripts encoding antioxidant enzymes, including SOD1 and SOD2, were elevated (Figure 7), suggesting the activation of compensatory antioxidant responses. These findings, together with elevated mtROS and NRF2 activation and translocation, suggest that Timeless depletion in human cells induces metabolic and redox imbalance characterized by reduced glycolytic capacity and increased reliance on oxidative metabolism.

Together, these findings support a model in which Timeless/Swi1 is functionally linked to mitochondrial homeostasis in both *S. pombe* and human cells, potentially linking replication stress response pathways to mitochondrial homeostasis. In addition to its role in genome integrity, our data indicate that Timeless/Swi1 is associated with the regulation of cellular metabolic state, and that its loss triggers conserved metabolic reprogramming characterized by suppression of glycolytic and anabolic pathways and increased reliance on oxidative metabolism. Given the well-established function of Timeless/Swi1 in maintaining genomic integrity, it is possible that at least some of the mitochondrial phenotypes observed in this study arise indirectly from broader genomic stress. At present, neither our data nor previous studies provide definitive evidence that Timeless/Swi1 or Rad52 localizes to mitochondria or directly regulates mitochondrial DNA metabolism. Future studies will be required to determine whether Timeless/Swi1 directly contributes to mitochondrial genome maintenance, regulates mitochondrial gene expression, or influences mitochondrial function indirectly through nuclear genome stress responses and associated signaling pathways. Elucidating these mechanisms will provide important insights into how nuclear and mitochondrial genome maintenance is coordinated and how their dysregulation contributes to disease.

## 5. Conclusions

This study identifies Timeless and its fission yeast ortholog Swi1 as previously unrecognized contributors to mitochondrial genome stability and function, extending their well-established roles in nuclear DNA replication and repair. The loss of Swi1 in fission yeast increases mitochondrial genome stress and instability but paradoxically induces a compensatory response characterized by an elevated mtDNA copy number, enhanced respiratory growth, and partial suppression of defects caused by impaired mitochondrial DNA polymerase γ, consistent with adaptive regulation of mitochondrial homeostasis under stress and a shift toward respiratory metabolism. In contrast, Timeless depletion in U-2 OS human cells reduces the mtDNA copy number, mitochondrial membrane potential, and mitochondrial mass, whereas TE-11 and Saos2 cells exhibit an increased mtDNA copy number, mitochondrial membrane potential, and mitochondrial mass, similar to the phenotypes observed in fission yeast. Notably, all human cell lines show elevated mitochondrial ROS, indicative of mitochondrial dysfunction. Transcriptomic analyses further reveal enrichment of oxidative phosphorylation and ROS-related pathways, supporting a metabolic shift toward increased reliance on mitochondrial respiration. Despite these divergent phenotypic outcomes, both systems activate conserved compensatory pathways involving mitochondrial and antioxidant responses, suggesting an evolutionarily conserved response to mitochondrial stress with mechanistically distinct consequences across species.

## Data Availability

All data supporting the findings of this study are available from the corresponding author upon reasonable request. The U-2 OS RNA-seq data have been deposited in the NCBI Gene Expression Omnibus (GEO) under accession number GSE339246. The *S. pombe* RNA-seq and ChIP-seq datasets have also been deposited in the NCBI GEO under accession numbers GSE342402 and GSE342403.

## Supplementary Materials

The following supporting information can be downloaded at: https://www.mdpi.com/article/10.3390/biom16081177/s1, Figure S1: GSEA enrichment plot for the glycolysis pathway in *S. pombe swi1*Δ cells. GSEA enrichment curve of RNA-seq data comparing *swi1*Δ and wild-type cells. The glycolysis gene set shows significant negative enrichment in *swi1*Δ cells, consistent with the pathway-level analyses presented in Figure 2B and supporting reduced glycolytic activity following Swi1 loss; Figure S2: GSEA enrichment plot for the oxidative phosphorylation pathway in *S. pombe swi1*Δ cells. GSEA enrichment curve of RNA-seq data comparing *swi1*Δ and wild-type cells. The oxidative phosphorylation gene set shows significant positive enrichment in *swi1*Δ cells, consistent with the pathway-level analyses presented in Figure 2C and supporting enhanced oxidative phosphorylation activity following Swi1 loss; Figure S3: GSEA enrichment plot for the oxidative phosphorylation pathway in U-2 OS osteosarcoma cells. GSEA enrichment curve of RNA-seq data comparing U-2 OS cells expressing Timeless shRNA and scrambled shRNA. The oxidative phosphorylation gene set shows significant positive enrichment in Timeless shRNA cells, consistent with the pathway-level analyses presented in Figure 7B and supporting enhanced oxidative phosphorylation activity following Timeless loss; Figure S4: GSEA enrichment plot for the reactive oxygen species pathway in U-2 OS osteosarcoma cells. GSEA enrichment curve of RNA-seq data comparing U-2 OS cells expressing Timeless shRNA and scrambled shRNA. The reactive oxygen species gene set shows significant positive enrichment in Timeless shRNA cells, consistent with the pathway-level analyses presented in Figure 7C and supporting enhanced reactive oxygen species activity following Timeless loss; Figure S5: GSEA heatmap for the oxidative phosphorylation pathway in U-2 OS osteosarcoma cells. GSEA heatmap of RNA-seq data comparing U-2 OS cells expressing Timeless shRNA and scrambled shRNA. The HALLMARK_OXIDATIVE_PHOSPHORYLATION gene set shows significant positive enrichment in Timeless shRNA cells. This extended dataset shows all leading-edge genes from the GSEA line plot and heatmap presented in Figure S3 and Figure 7B; Figure S6: Flow cytometry histogram for mitochondrial superoxide measurement in U-2 OS osteosarcoma cells. Histogram of flow cytometry data comparing mitochondrial superoxide levels in U-2 OS cells treated with Timeless siRNAs and control siRNA. The histograms show a modest shift and increase in mitochondrial superoxide levels in Timeless siRNA cells; Figure S7: Flow cytometry histogram for mitochondrial potential measurement in U-2 OS osteosarcoma cells. Histogram of flow cytometry data comparing mitochondrial membrane potential in U-2 OS cells treated with Timeless siRNAs and control siRNA. The histograms show a modest shift and decrease in mitochondrial potential in Timeless siRNA cells; Figure S8: Flow cytometry histogram for mitochondrial content measurement in U-2 OS osteosarcoma cells. Histogram of flow cytometry data comparing mitochondrial content in U-2 OS cells treated with Timeless siRNAs and control siRNA. The histograms show a modest shift and decrease in mitochondrial content in Timeless siRNA cells, consistent with mtDNA qPCR analyses presented in Figure 3D; Figure S9: Flow cytometry histogram for mitochondrial content measurement in Saos2 osteosarcoma cells. Histogram of flow cytometry data comparing mitochondrial content in Saos2 cells expressing Timeless shRNAs and scrambled shRNA. The histograms show a modest shift and increase in mitochondrial content in Timeless shRNA cells, consistent with mtDNA qPCR analyses presented in Figure 5B; Figure S10: Flow cytometry histogram for mitochondrial potential measurement in Saos2 osteosarcoma cells. Histogram of flow cytometry data comparing mitochondrial membrane potential in U-2 OS cells expressing Timeless shRNAs and scrambled shRNA. The histograms show no significant change in mitochondrial potential in Timeless shRNA cells; Figure S11: Flow cytometry histogram for mitochondrial superoxide measurement in Saos2 osteosarcoma cells. Histogram of flow cytometry data comparing mitochondrial superoxide levels in Saos2 cells expressing Timeless shRNAs and scrambled shRNA. The histograms show no significant change in mitochondrial superoxide levels in Timeless shRNA cells; Figure S12: Flow cytometry histogram for mitochondrial content measurement in TE-11 ESCC cells. Histogram of flow cytometry data comparing mitochondrial content in TE-11 cells expressing Timeless shRNAs and scrambled shRNA. The histograms show a modest shift and increase in mitochondrial content in Timeless shRNA cells, consistent with mtDNA qPCR analyses presented in Figure 5B; Figure S13: Flow cytometry histogram for mitochondrial potential measurement in TE-11 ESCC cells. Histogram of flow cytometry data comparing mitochondrial membrane potential in TE-11 cells expressing Timeless siRNAs and control siRNA. The histograms show a modest shift and increase in mitochondrial potential in Timeless siRNA cells; Figure S14: Flow cytometry histogram for mitochondrial superoxide measurement in TE-11 osteosarcoma cells. Histogram of flow cytometry data comparing mitochondrial superoxide levels in TE-11 cells expressing Timeless siRNAs and control siRNA. The histograms show a modest shift and increase in mitochondrial superoxide levels in Timeless siRNA cells; File S1: Original images of Western blotting results.

## Abbreviations

The following abbreviations are used in this manuscript:

DTT	Dithiothreitol
FBS	Fetal bovine serum
FE	Fold enrichment
IGV	Integrated genomics viewer
KEGG	Kyoto Encyclopedia of Genes and Genomes
mtDNA	Mitochondrial DNA
mtROS	Mitochondrial reactive oxygen species
PBS	Phosphate-buffered saline
PI	Propidium iodide
Pol γ	DNA polymerase γ
ROS	Reactive oxygen species
TFAM	Mitochondrial transcription factor A
TMRE	Tetramethylrhodamine ethyl ester perchlorate
YEGlyE	Yeast extract, glycerol, and ethanol
YES	Yeast extract and supplements

## Appendix A

**Table A1:** *S. pombe* strains used in this study.

Figure	Strain	Genotype
Figure 1A, B	Y4250	h+ rad52-12PK:kanMX6 leu1-32 ura4-D18
Figure 1A, B	Y4256	h+ swi1::kanMX6 rad52-12PK:natMX6 leu1-32 ura4-D18
Figure 1C	Y0001	h- leu1-32 ura4-D18
Figure 1C	Y0002	h+ leu1-32 ura4-D18
Figure 1C	Y5272	h+ kanMX6-P81nmt-FLAG:pog1 leu1-32 ura4-D18
Figure 1C	Y5273	h+ kanMX6-P81nmt-FLAG:pog1 leu1-32 ura4-D18
Figure 1D	Y0001	h- leu1-32 ura4-D18
Figure 1D	Y1679	h- swi1::natMX6 leu1-32 ura4-D18
Figure 1D	Y5271	h- kanMX6-P81nmt-FLAG:pog1 leu1-32 ura4-D18
Figure 1D	Y5275	h- swi1::natMX6 kanMX6-Pnmt81-FLAG-pog1 leu1-31 ura4-D18
Figure 2	Y0001	h- leu1-32 ura4-D18
Figure 2	Y1679	h- swi1::natMX6 leu1-32 ura4-D18
Figure 2	Y5273	h+ kanMX6-P81nmt-FLAG:pog1 leu1-32 ura4-D18
Figure 2	Y5274	h+ swi1::natMX6 kanMX6-Pnmt81-FLAG-pog1 leu1-31 ura4-D18
Figure 2	Y5271	h- kanMX6-P81nmt-FLAG:pog1 leu1-32 ura4-D18
Figure 2	Y5275	h- swi1::natMX6 kanMX6-Pnmt81-FLAG-pog1 leu1-31 ura4-D18
Figure 5B	Y4250	h+ rad52-12PK:kanMX6 leu1-32 ura4-D18
Figure 5B	Y4256	h+ swi1::kanMX6 rad52-12PK:natMX6 leu1-32 ura4-D18

**Table A2:** Oligonucleotide primers used for *S. pombe* strain construction.

No.	Name	Sequence
P1878	Ppog1-5′	CTG TTT ATA CTT GCT GAT TTC TC
P1885	Ppog1-3′dep module-N	GTT TAA ACG AGC TCG AAT TCG AGC AAT GGA CGC CCA ACT GGA ATT TAC
P1887	pog1NT-3′	CTT TAT TCA GTA GTT GGT GCT TCG
P1893	pog1NT-5′depFLmodule	CTA TAA GGA CGA TGA TGA TAA AGG AGG CGG AAT GTT CTA CAA AGC TTG TCC AAG TAC

**Table A3:** Oligonucleotide primers used for mtDNA copy number assay.

No.	Name	Sequence
P1850	ND1-F	CCC TAA AAC CCG CCA CAT CT
P1851	ND1-R	GAG CGA TGG TGA GAG CTA AGG T
P1852	HGB-1	GTG CAC CTG ACT CCT GAG GAG A
P1853	HGB-2	CCT TGA TAC CAA CCT GCC CAG
P1854	cox1-RT-F	TGG ACG GTA TAT CCA CCA CT
P1855	cox1-RT-R	GTC GCT ATT AAA TTT ACT GAT CC
P1868	mtF3163	GCC TTC CCC CGT AAA TGA TA
P1869	mtR3260	TTA TGC GAT TAC CGG GCT CT
P1874	atp9-RT-F	GGT GCT GGT GTT GGT ATT GGA
P1875	atp9-RT-R	ACC TGT AGC TTC TGT TAA GGC G
P1876	Spo12-F	TCG GCT CTA AGA AGG TAT CTG TAT C
P1877	Spo12-R	AGT ACC AGA TCT GCC TGA GTA GTT G
P1889	mtND4-F	GCT CCA TCT GCC TAC GAC AA
P1890	mtND4-R	GCT TCA GGG GGT TTG GAT GA
P1891	mtCO1-F	CGC CGA CCG TTG ACT ATT CT
P1892	mtCO1-R	CGG CTC GAA TAA GGA GGC TT
