# Supplementary material for: Evolutionarily Conserved but Mechanistically Distinct Mitochondrial Responses to Loss of Timeless/Swi1
Source: Biomolecules. 2026 Aug 12;16(8):1177. doi: 10.3390/biom16081177 (PMC13511166; doi:10.3390/biom16081177)
Supplement: Supplementary file 1 [file biomolecules-16-01177-s001.zip › biomolecules-4335153-supplementary.pdf]

Supplementary Figures

S1

S. pombe

Glycolysis/Gluconeogenesis

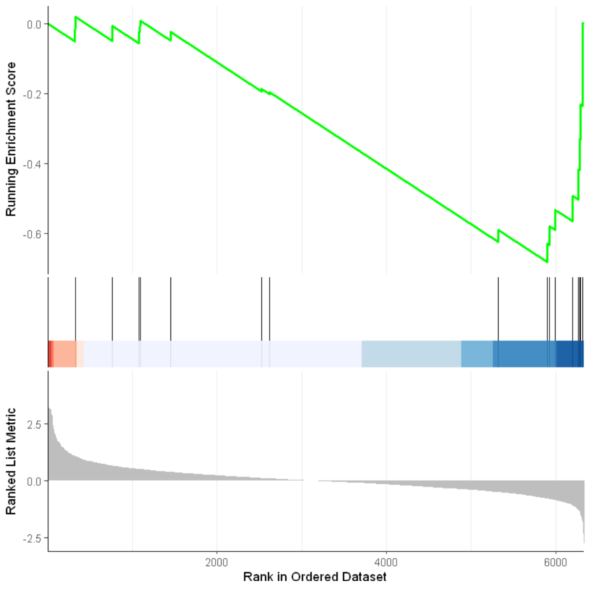

S2

S. pombe

Oxidative Phosphorylation

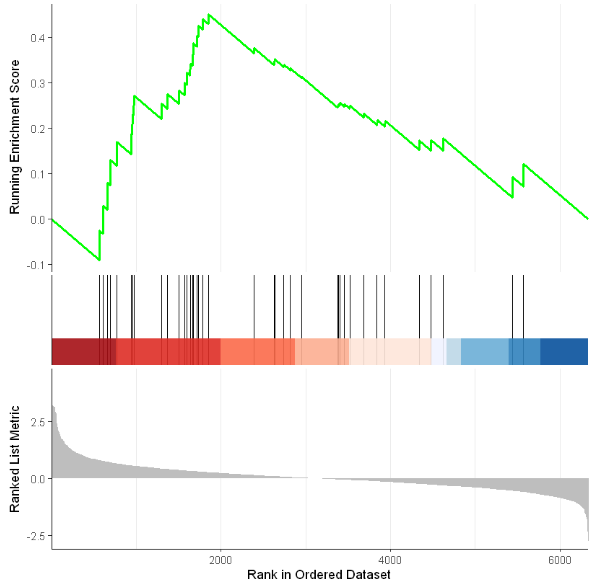

S3

U-2 OS

Oxidative Phosphorylation

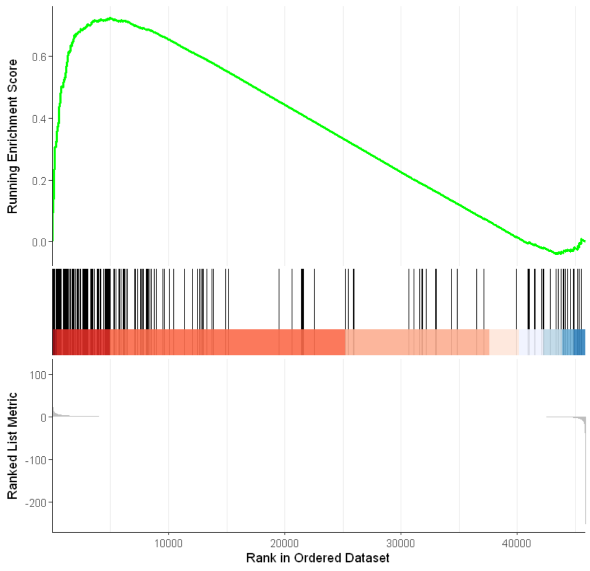

S4

U-2 OS

Reactive Oxygen Species

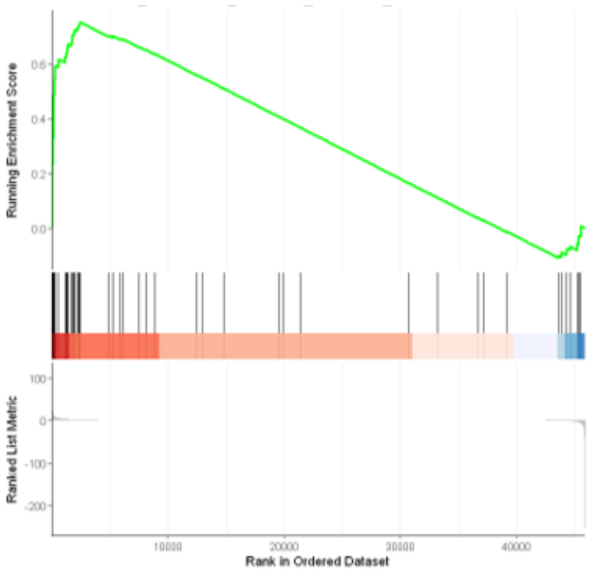

Supplemental Figures

S5

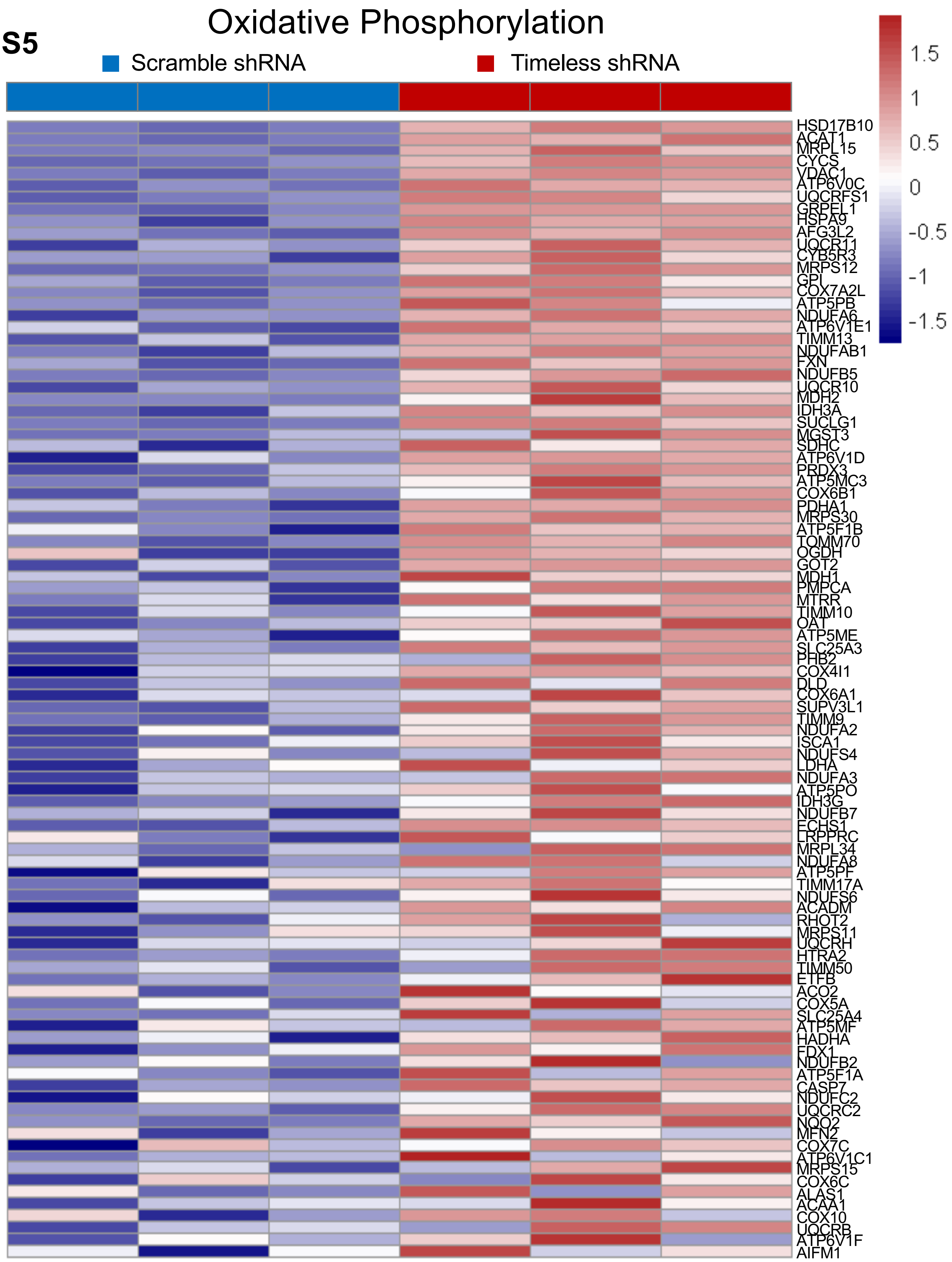

S6

U-2 OS Mitochondrial
Superoxide

siCtrl

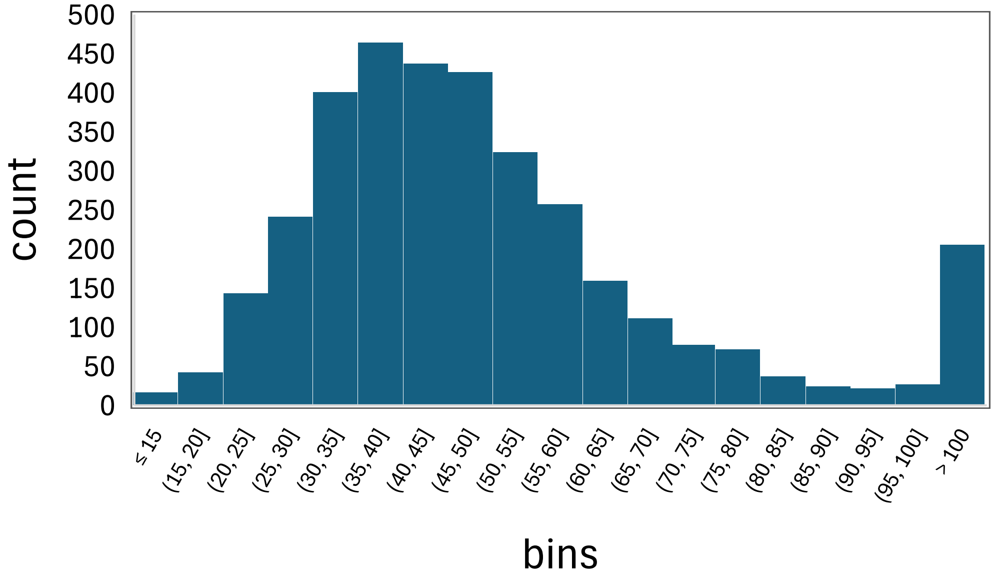

siTimeless#1

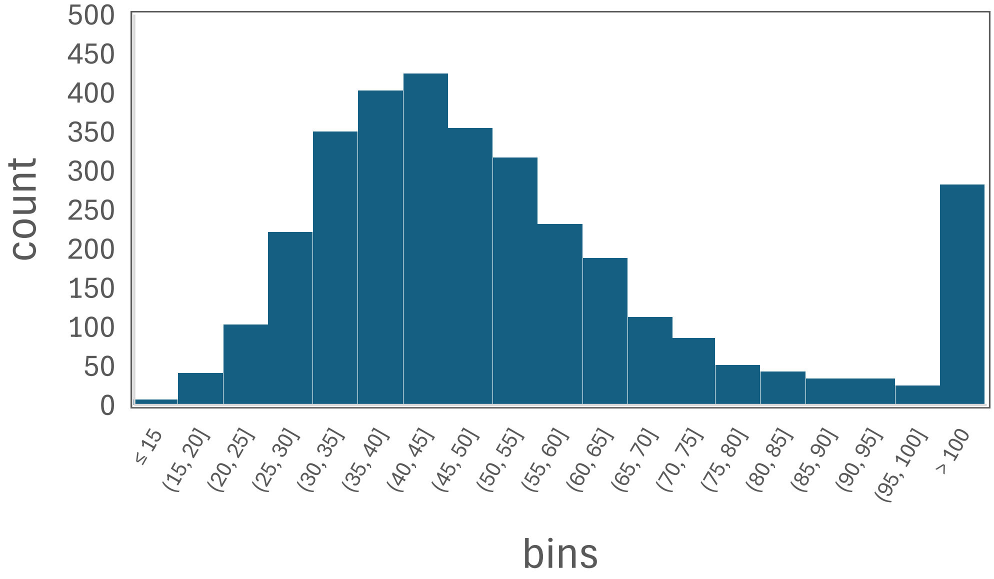

siTimeless#2

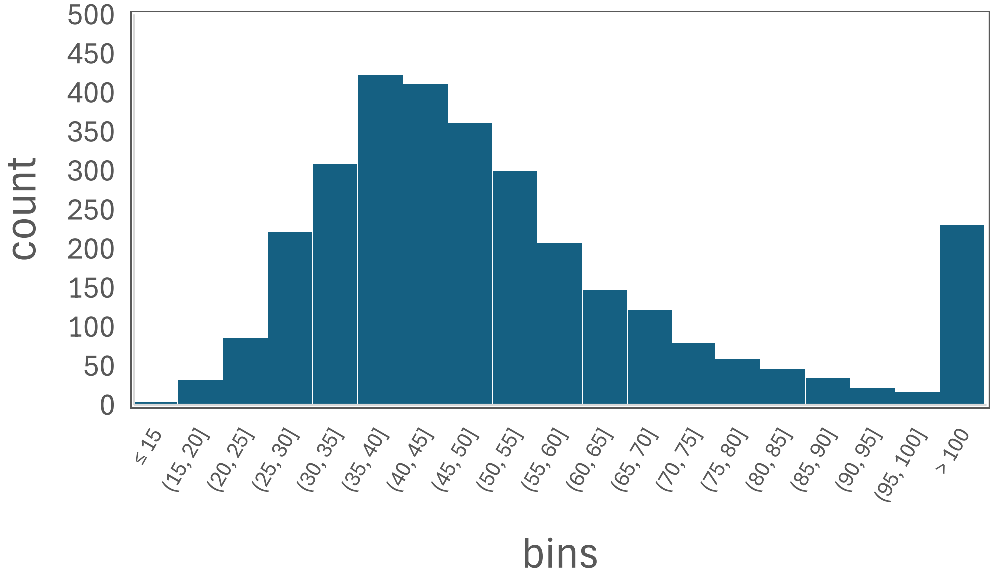

S7

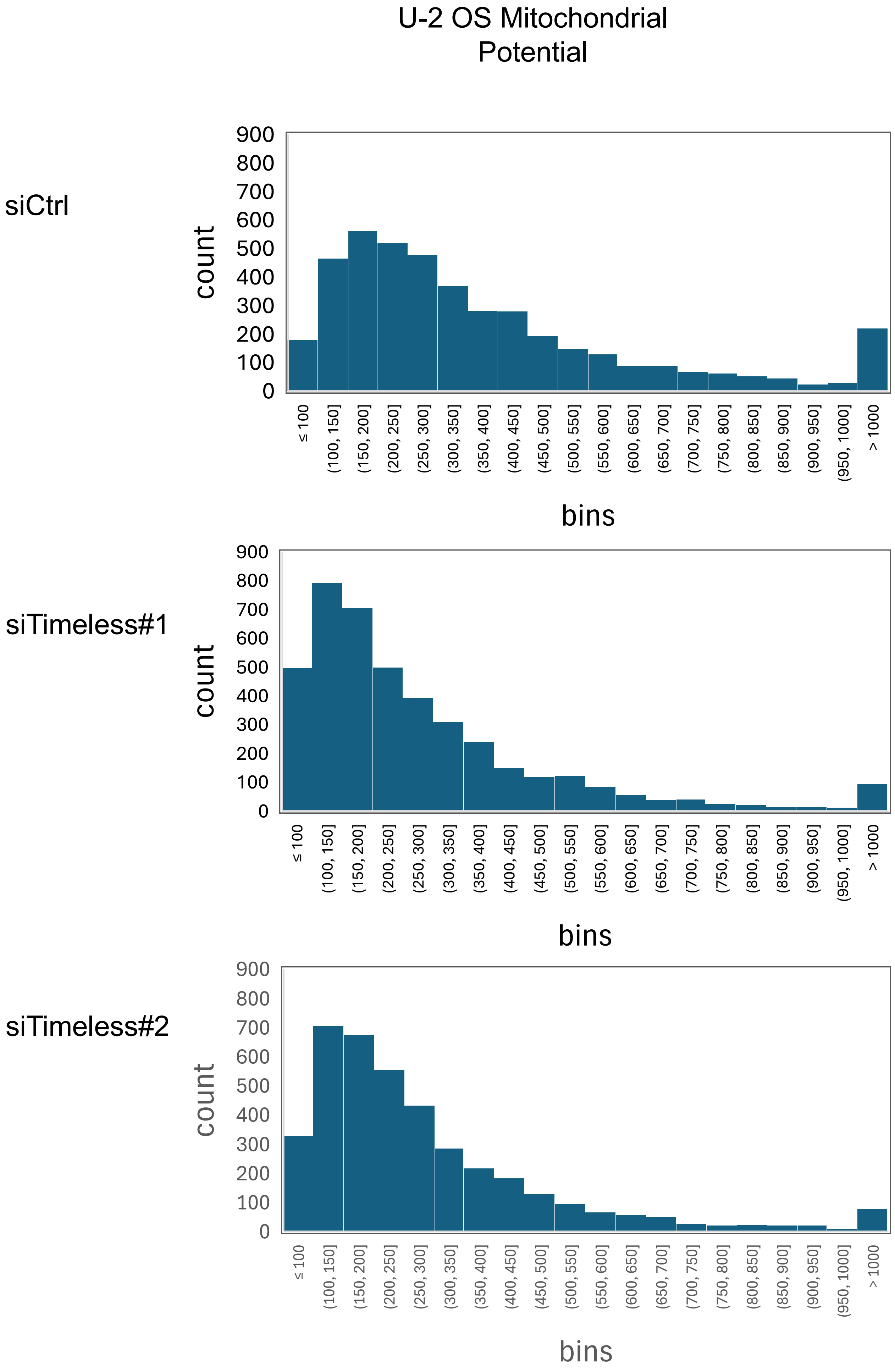

U-2 OS Mitochondrial Potential
siCtrl
siTimeless#1
siTimeless#2
count
bins
0
100
200
300
400
500
600
700
800
900
≤ 100
(100, 150]
(150, 200]
(200, 250]
(250, 300]
(300, 350]
(350, 400]
(400, 450]
(450, 500]
(500, 550]
(550, 600]
(600, 650]
(650, 700]
(700, 750]
(750, 800]
(800, 850]
(850, 900]
(900, 950]
(950, 1000]
> 1000

S8

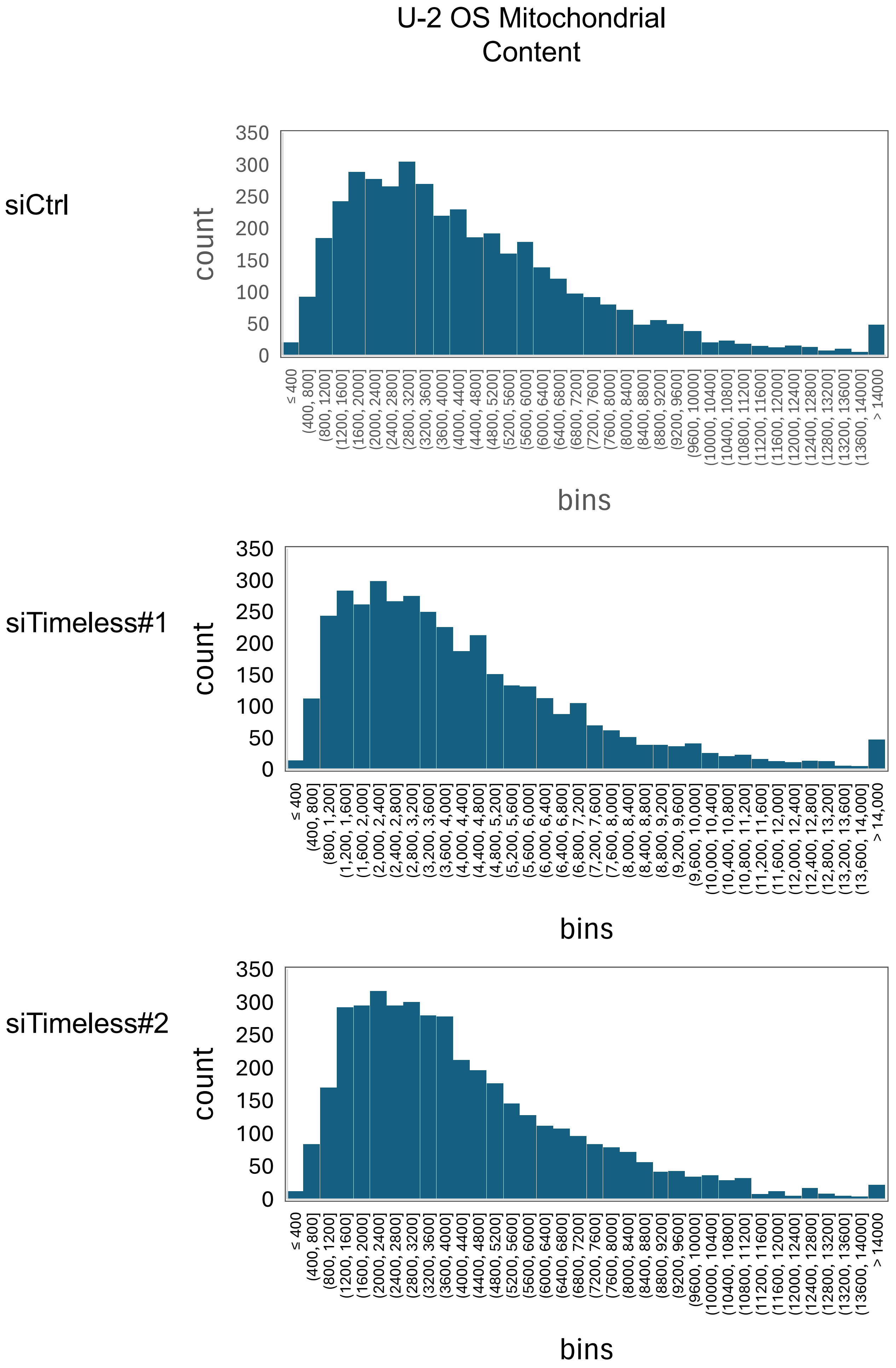

U-2 OS Mitochondrial Content
siCtrl
count
350
300
250
200
150
100
50
0
≤ 400
(400, 800]
(800, 1200]
(1200, 1600]
(1600, 2000]
(2000, 2400]
(2400, 2800]
(2800, 3200]
(3200, 3600]
(3600, 4000]
(4000, 4400]
(4400, 4800]
(4800, 5200]
(5200, 5600]
(5600, 6000]
(6000, 6400]
(6400, 6800]
(6800, 7200]
(7200, 7600]
(7600, 8000]
(8000, 8400]
(8400, 8800]
(8800, 9200]
(9200, 9600]
(9600, 10000]
(10000, 10400]
(10400, 10800]
(10800, 11200]
(11200, 11600]
(11600, 12000]
(12000, 12400]
(12400, 12800]
(12800, 13200]
(13200, 13600]
(13600, 14000]
> 14000
bins
siTimeless#1
count
350
300
250
200
150
100
50
0
≤ 400
(400, 800]
(800, 1,200]
(1,200, 1,600]
(1,600, 2,000]
(2,000, 2,400]
(2,400, 2,800]
(2,800, 3,200]
(3,200, 3,600]
(3,600, 4,000]
(4,000, 4,400]
(4,400, 4,800]
(4,800, 5,200]
(5,200, 5,600]
(5,600, 6,000]
(6,000, 6,400]
(6,400, 6,800]
(6,800, 7,200]
(7,200, 7,600]
(7,600, 8,000]
(8,000, 8,400]
(8,400, 8,800]
(8,800, 9,200]
(9,200, 9,600]
(9,600, 10,000]
(10,000, 10,400]
(10,400, 10,800]
(10,800, 11,200]
(11,200, 11,600]
(11,600, 12,000]
(12,000, 12,400]
(12,400, 12,800]
(12,800, 13,200]
(13,200, 13,600]
(13,600, 14,000]
> 14,000
bins
siTimeless#2
count
350
300
250
200
150
100
50
0
≤ 400
(400, 800]
(800, 1200]
(1200, 1600]
(1600, 2000]
(2000, 2400]
(2400, 2800]
(2800, 3200]
(3200, 3600]
(3600, 4000]
(4000, 4400]
(4400, 4800]
(4800, 5200]
(5200, 5600]
(5600, 6000]
(6000, 6400]
(6400, 6800]
(6800, 7200]
(7200, 7600]
(7600, 8000]
(8000, 8400]
(8400, 8800]
(8800, 9200]
(9200, 9600]
(9600, 10000]
(10000, 10400]
(10400, 10800]
(10800, 11200]
(11200, 11600]
(11600, 12000]
(12000, 12400]
(12400, 12800]
(12800, 13200]
(13200, 13600]
(13600, 14000]
> 14000
bins

S9

Saos2 Mitochondrial Content

shScr

shTimeless#3

shTimeless#4

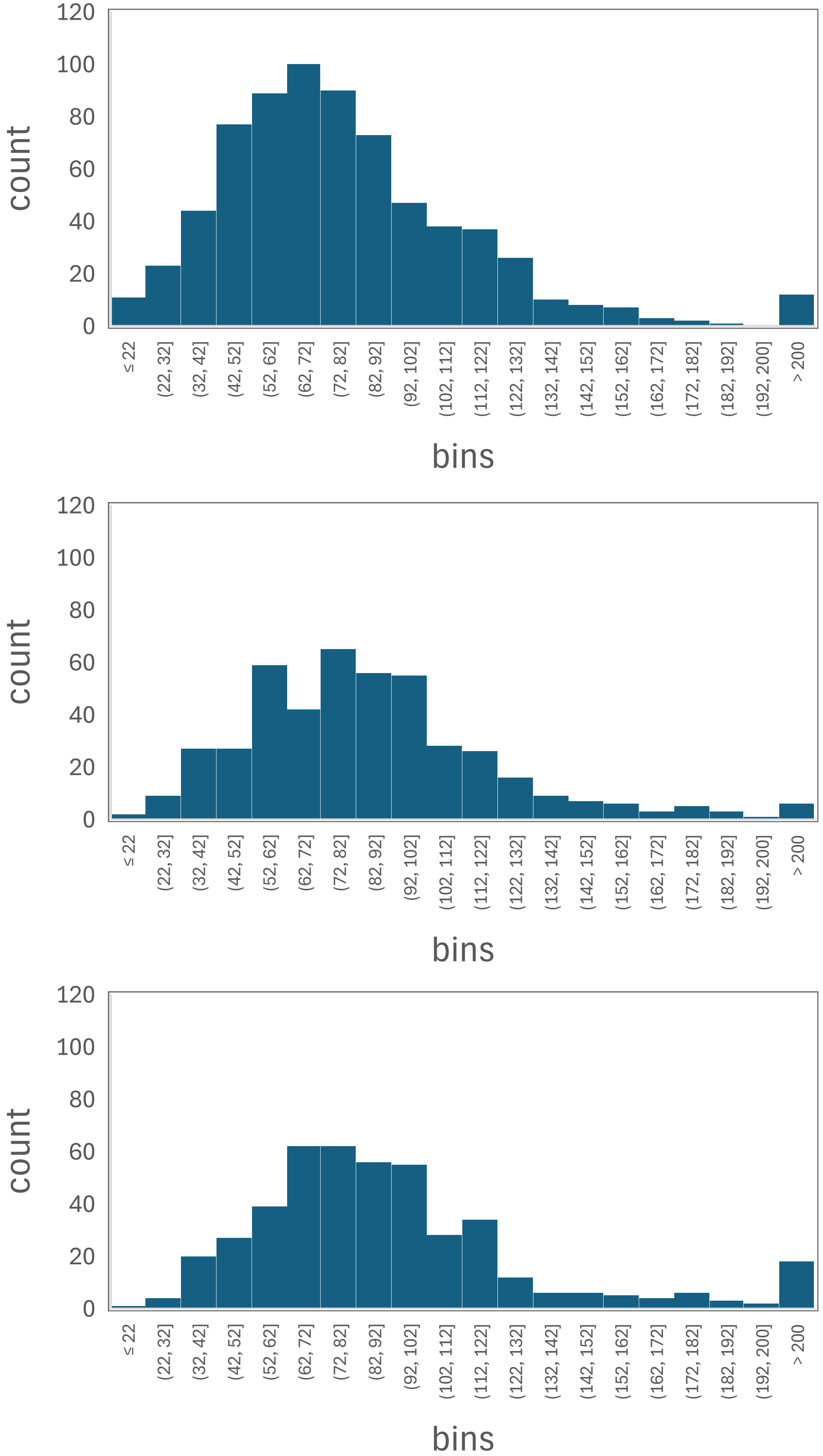

S10

Saos2 Mitochondrial
Potential

shScr

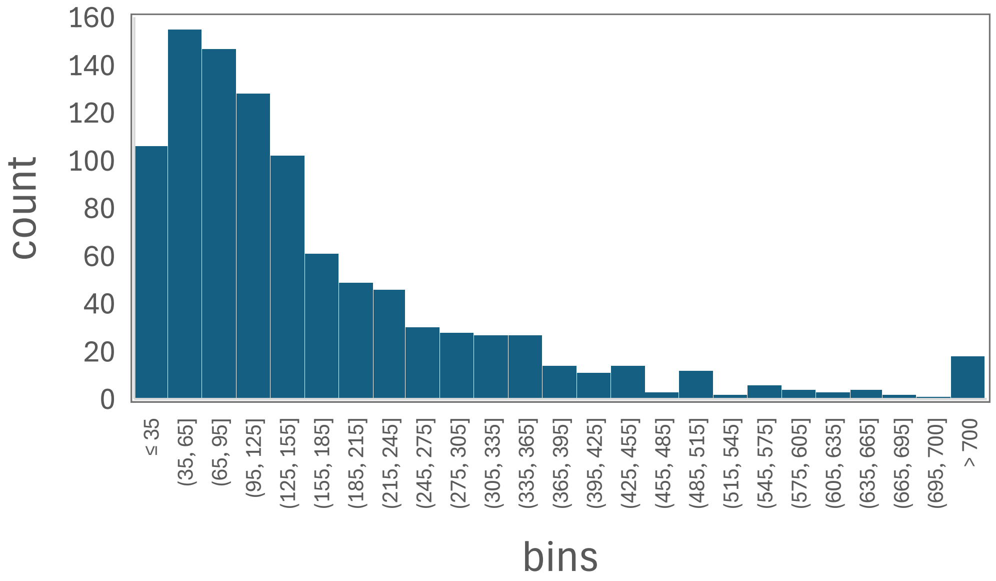

shTimeless#3

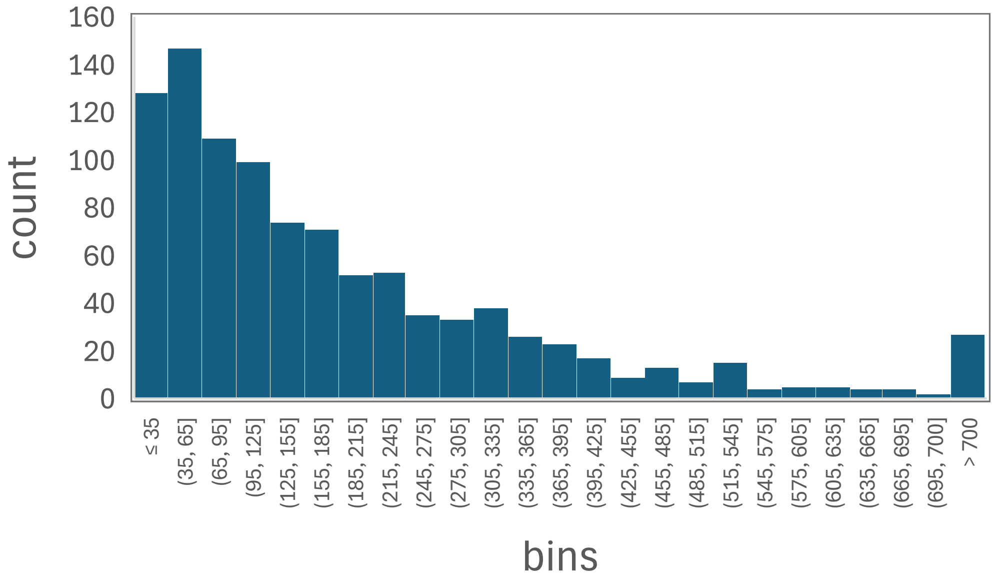

shTimeless#4

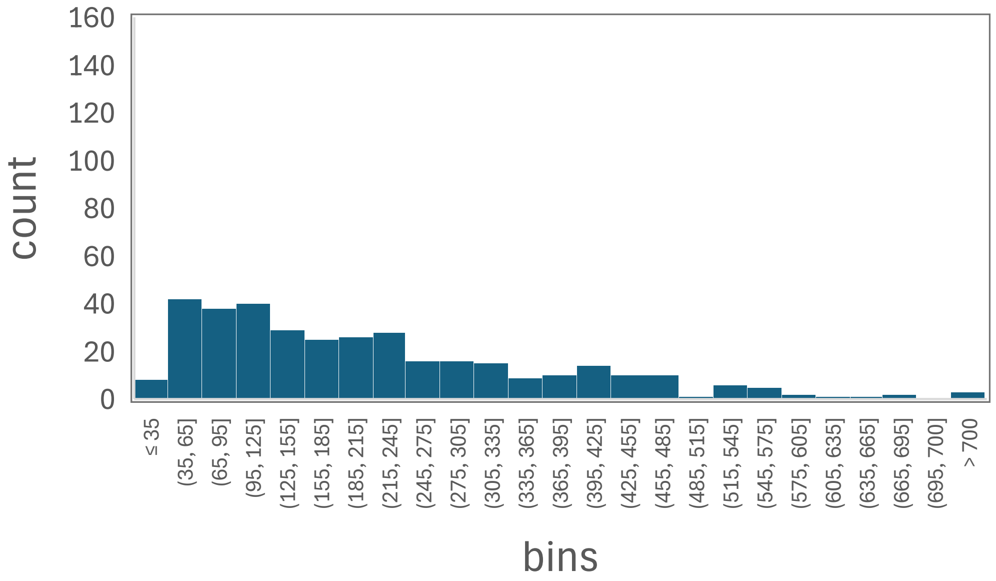

S11

Saos2 Mitochondrial
Superoxide

shScr

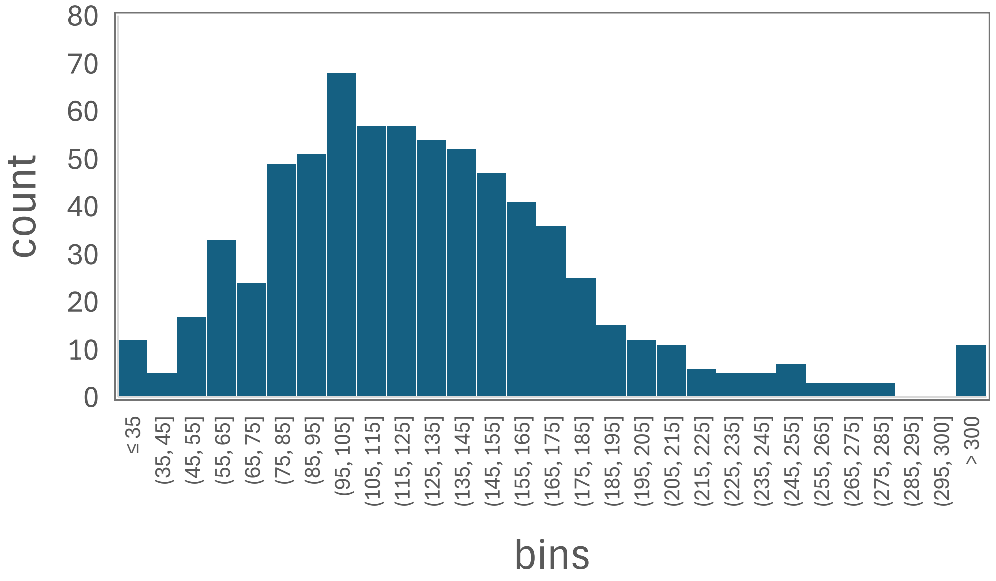

shTimeless#3

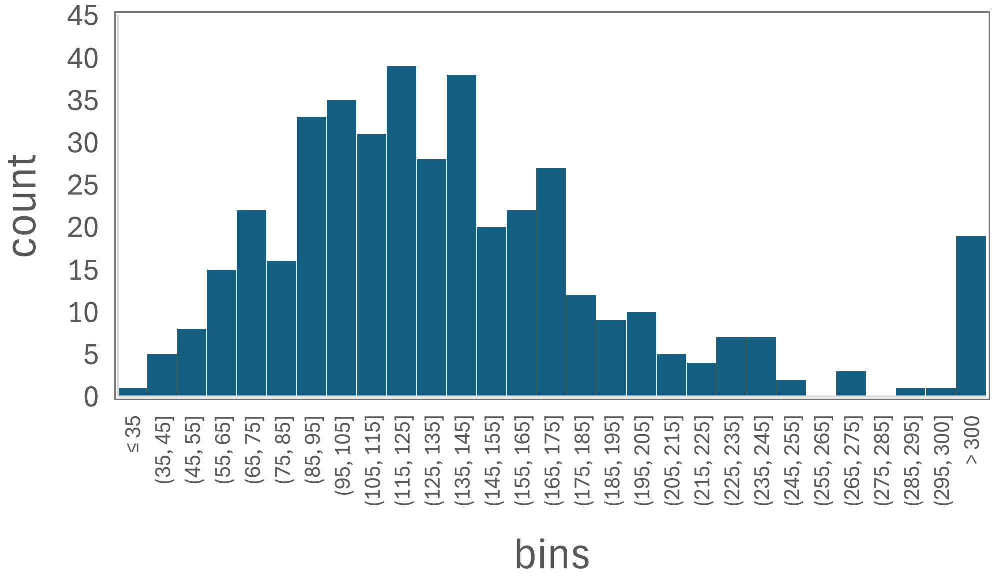

shTimeless#4

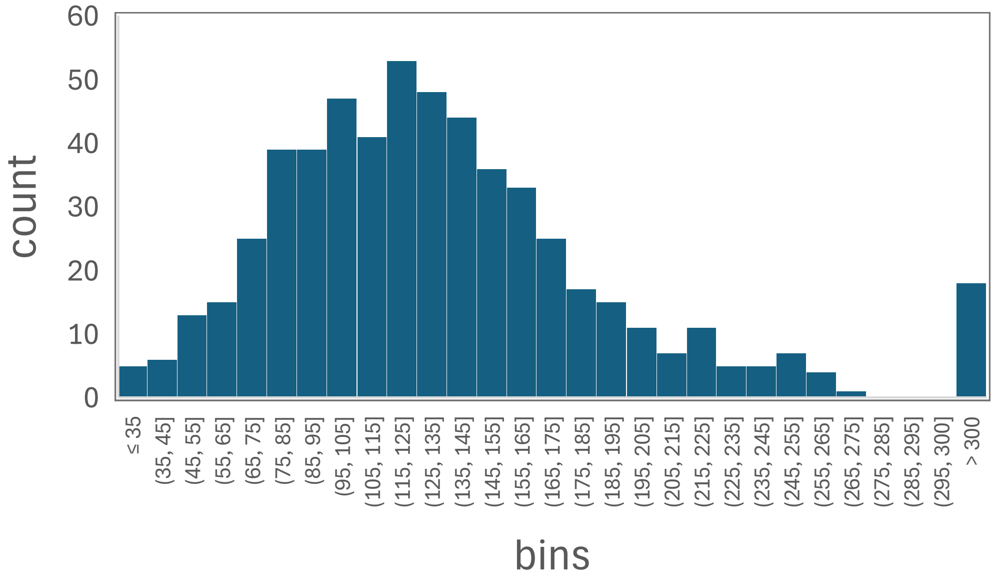

S12

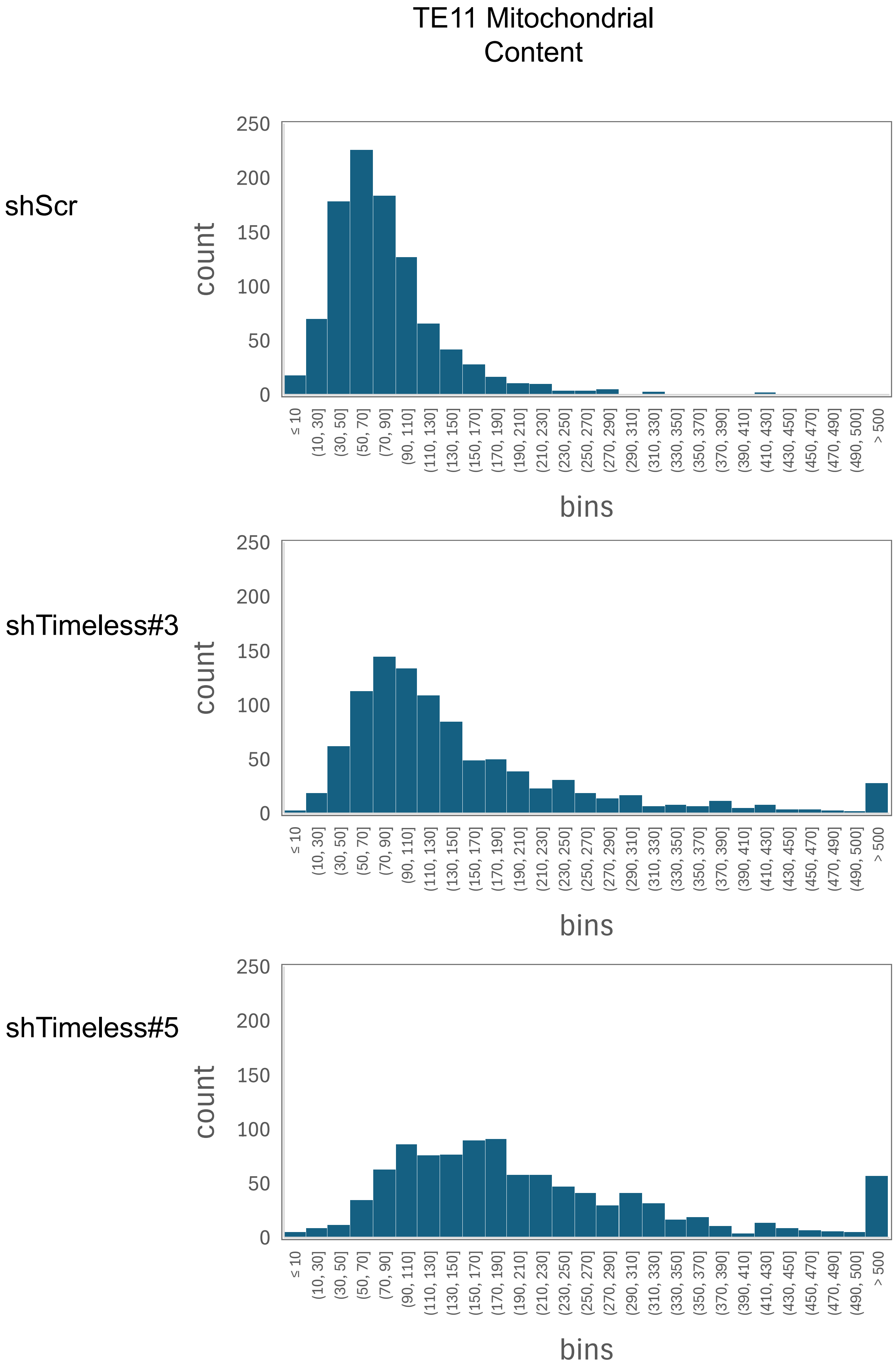

TE11 Mitochondrial Content
shScr
shTimeless#3
shTimeless#5
count
bins
0
50
100
150
200
250
≤ 10
(10, 30]
(30, 50]
(50, 70]
(70, 90]
(90, 110]
(110, 130]
(130, 150]
(150, 170]
(170, 190]
(190, 210]
(210, 230]
(230, 250]
(250, 270]
(270, 290]
(290, 310]
(310, 330]
(330, 350]
(350, 370]
(370, 390]
(390, 410]
(410, 430]
(430, 450]
(450, 470]
(470, 490]
(490, 500]
> 500

S13

TE11 Mitochondrial Potential

shScr

shTimeless#3

shTimeless#5

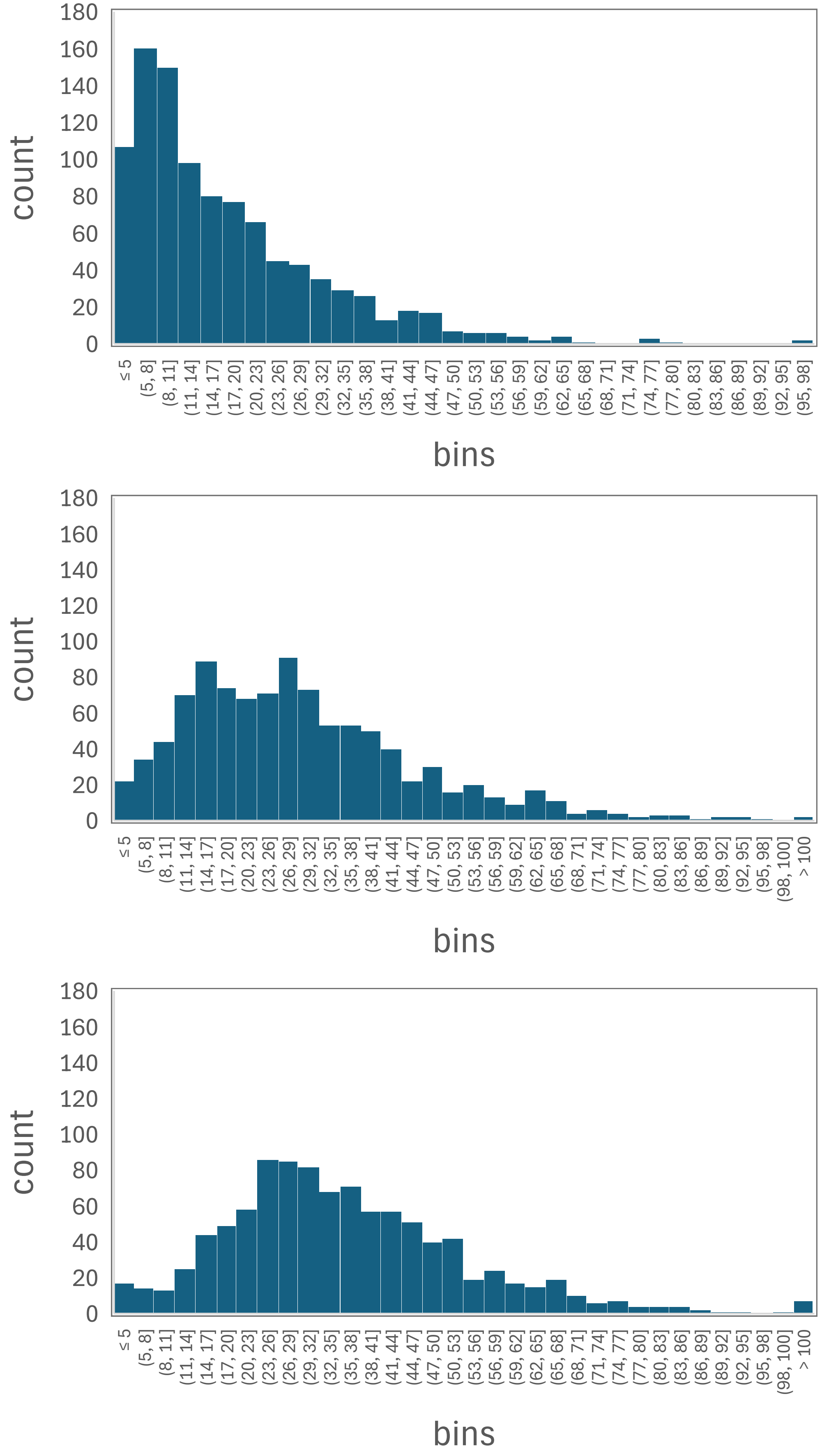

S14

TE11 Mitochondrial
Superoxide

shScr

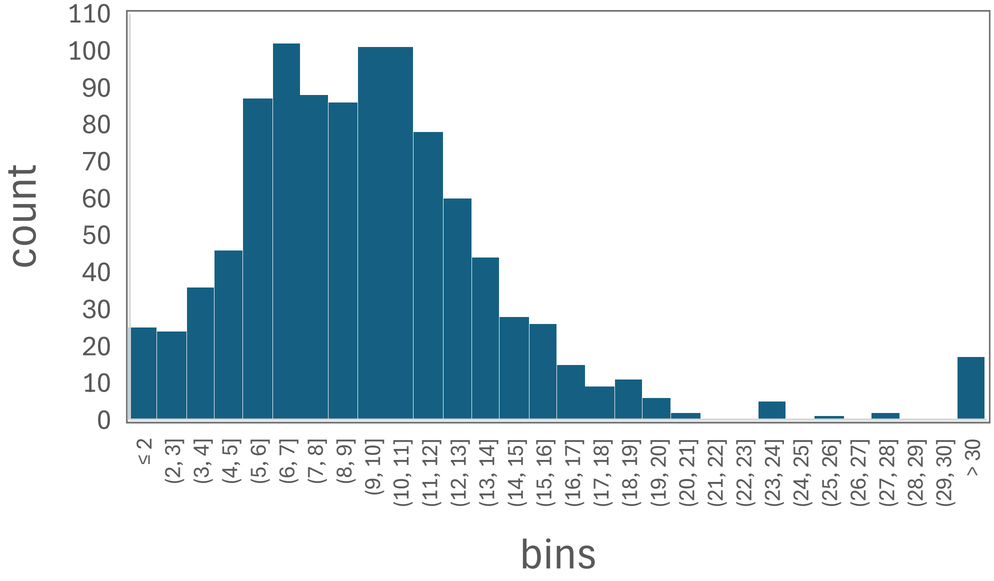

shTimeless#3

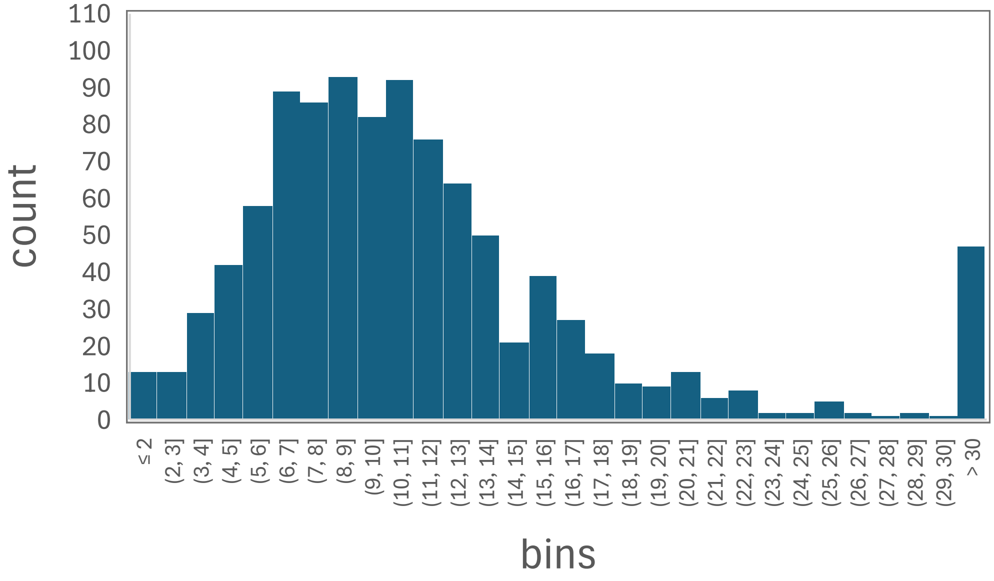

shTimeless#5

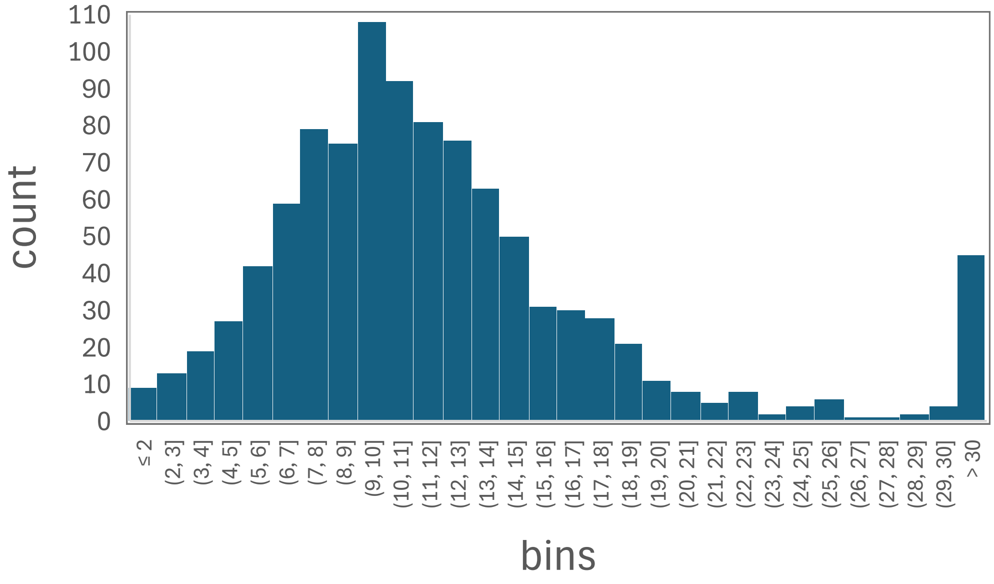

File S1

Uncropped Western blot images for Figure 1D

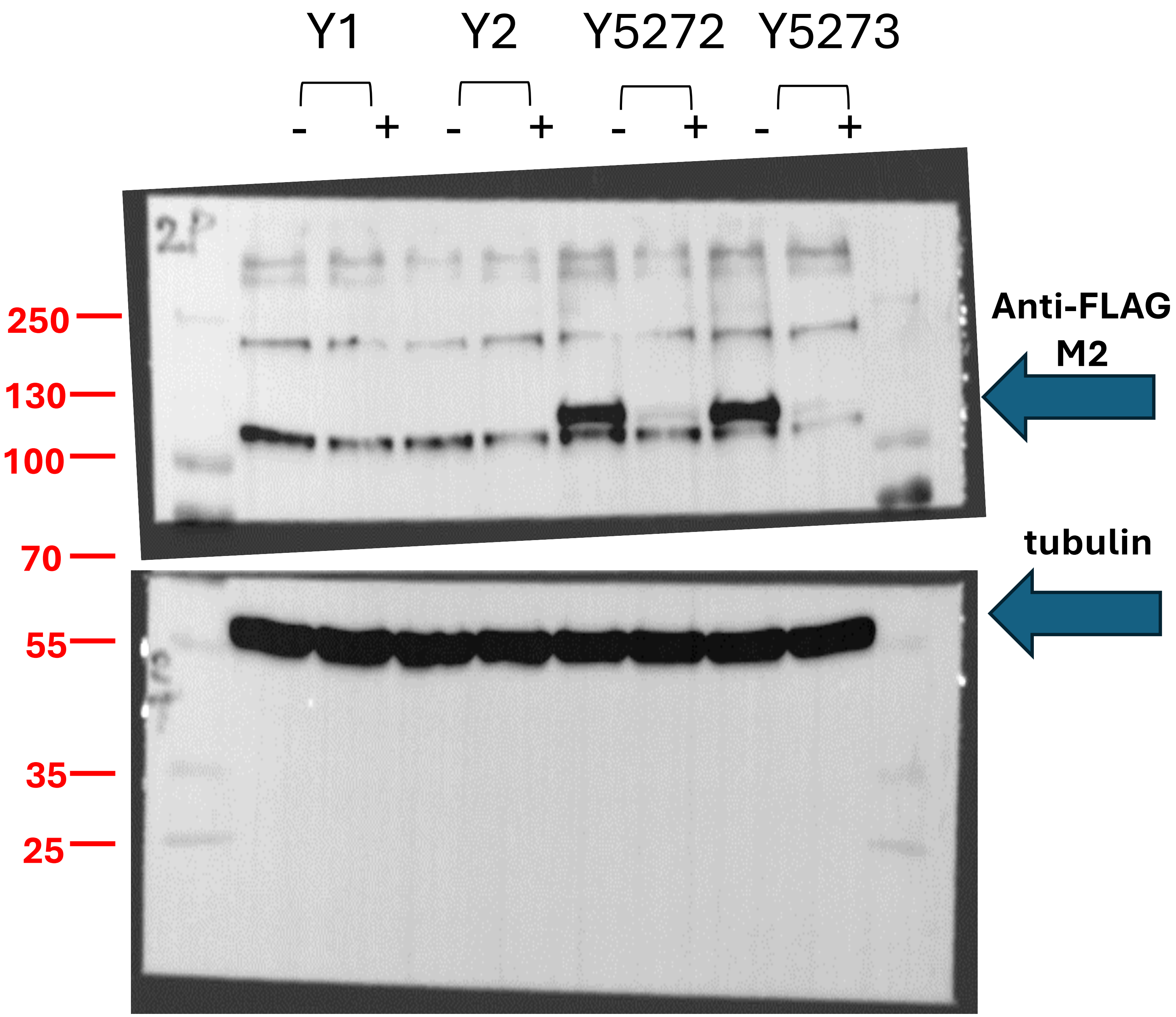

3FLAG Pog1 ratios: 1.235294, 0.296875, 0.137255, 0.163636, 8.260163, 0.656, 9.811966, 0.030928

Uncropped Western blot images for Figure 3A

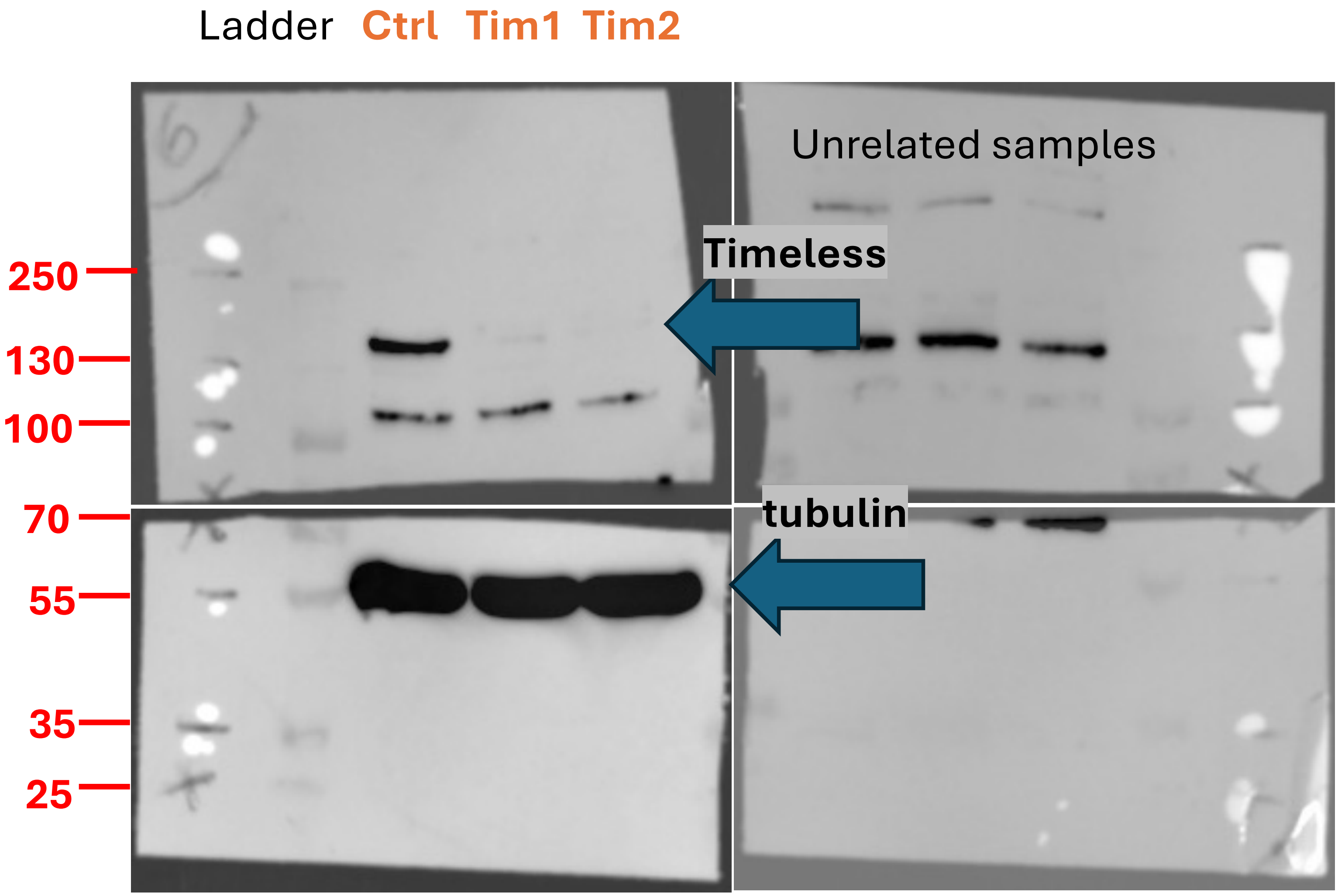

Timeless intensity ratios: 0.65288, 0.024723, 0.01213

Uncropped Western blot images for Figure 3B

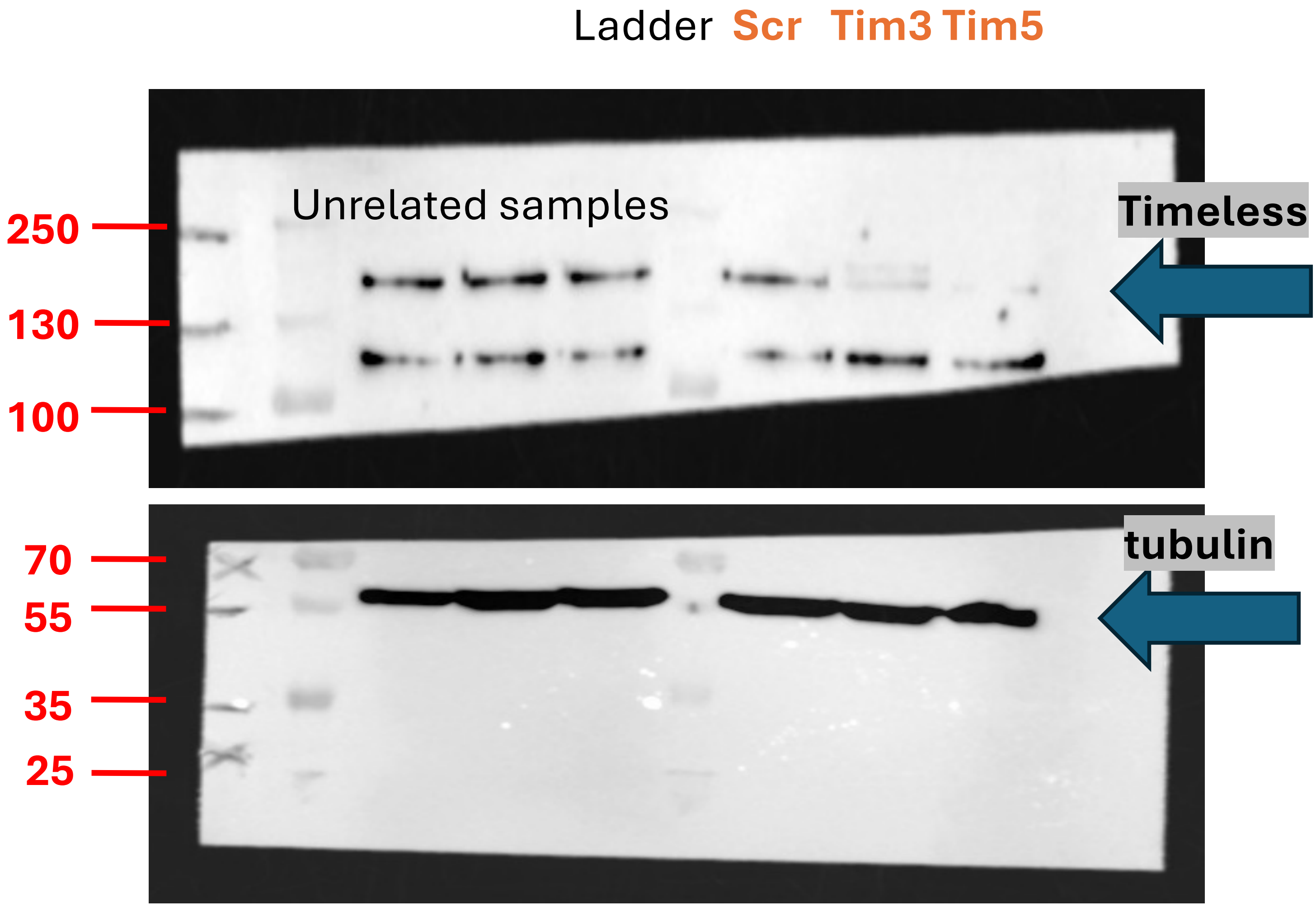

Timeless intensity ratios: 0.379969, 0.083401, 0.040037

Uncropped Western blot images for Figure 5A, Saos-2

Low exposure image for tubulin detection

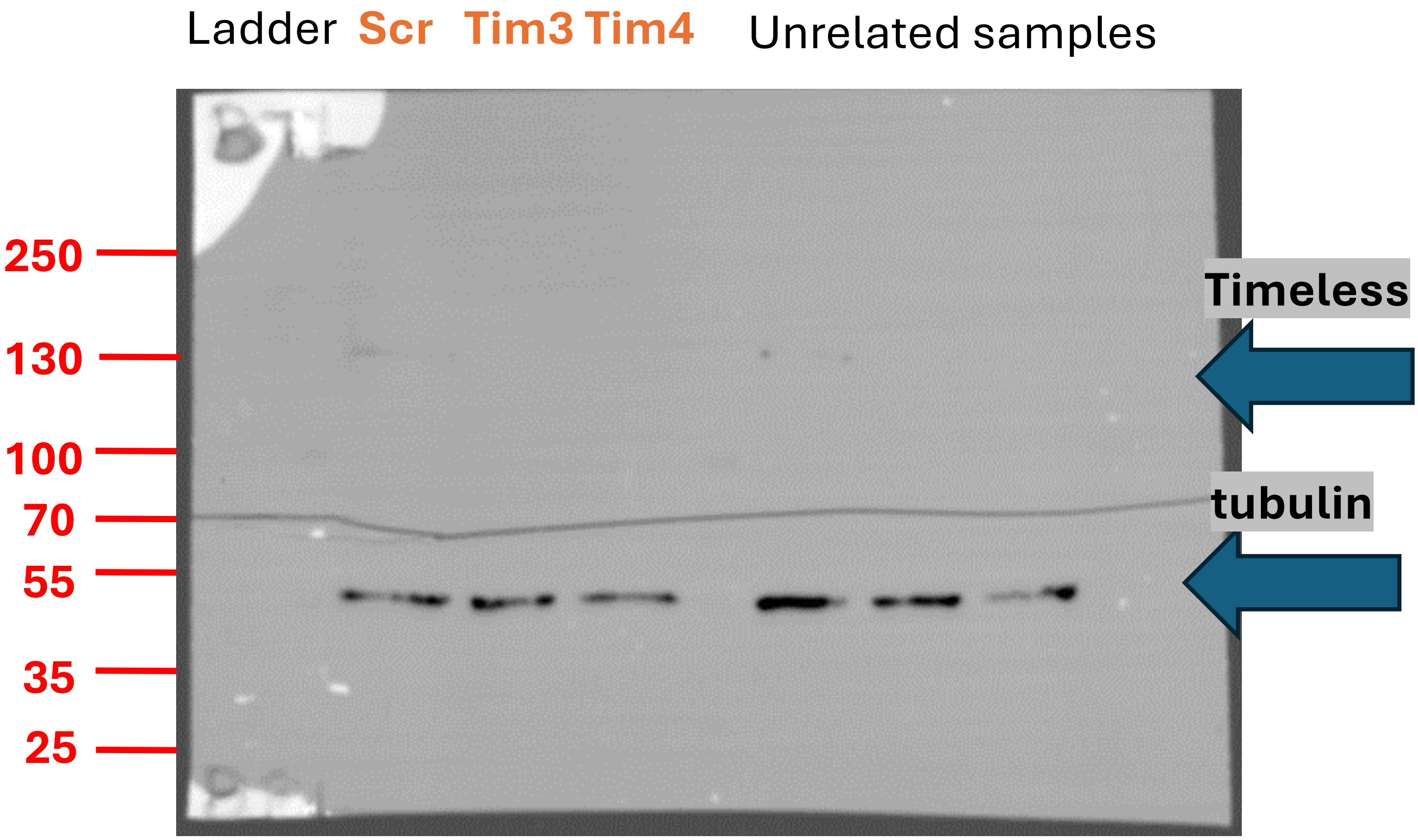

Uncropped Western blot images for Figure 5A, Saos-2

High exposure image for Timeless detection

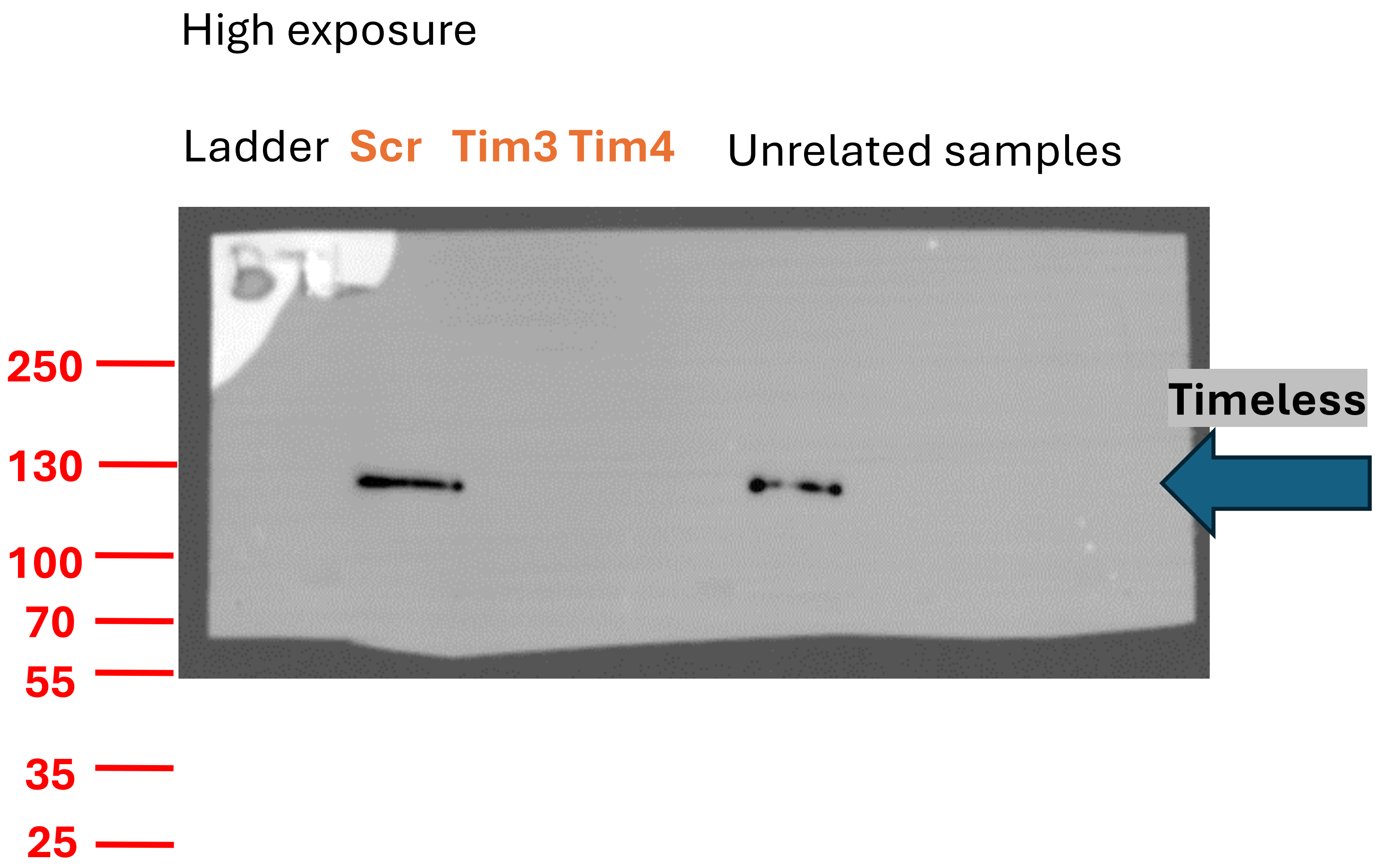

Timeless intensity ratios: 1.33452, 0, 0.087179

Uncropped Western blot images for Figure 5A , TE-11

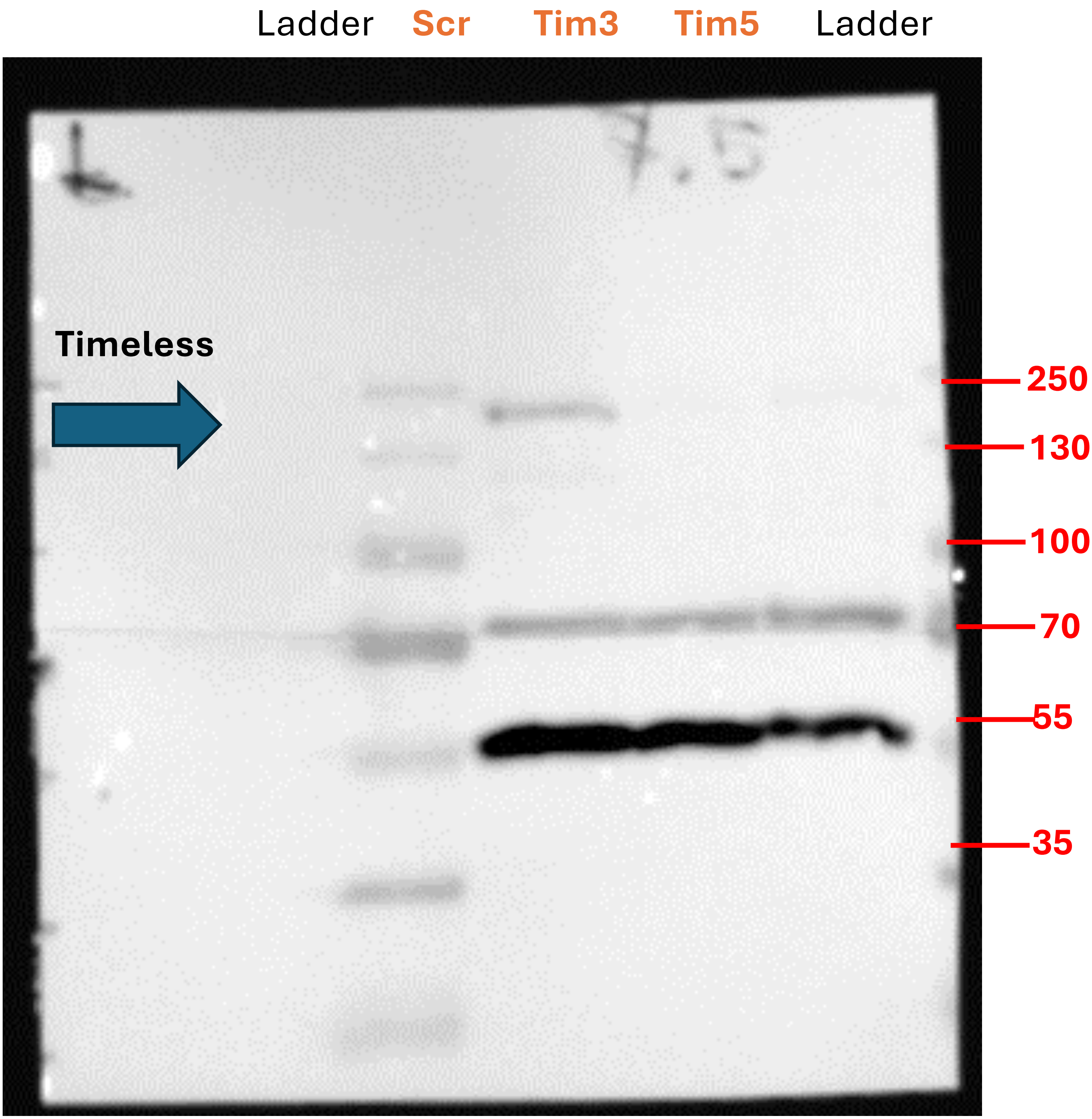

Timeless intensity ratios: 1.170475, 0.03333, 0.02286

Uncropped Western blot images for Figure 5A , TE-11

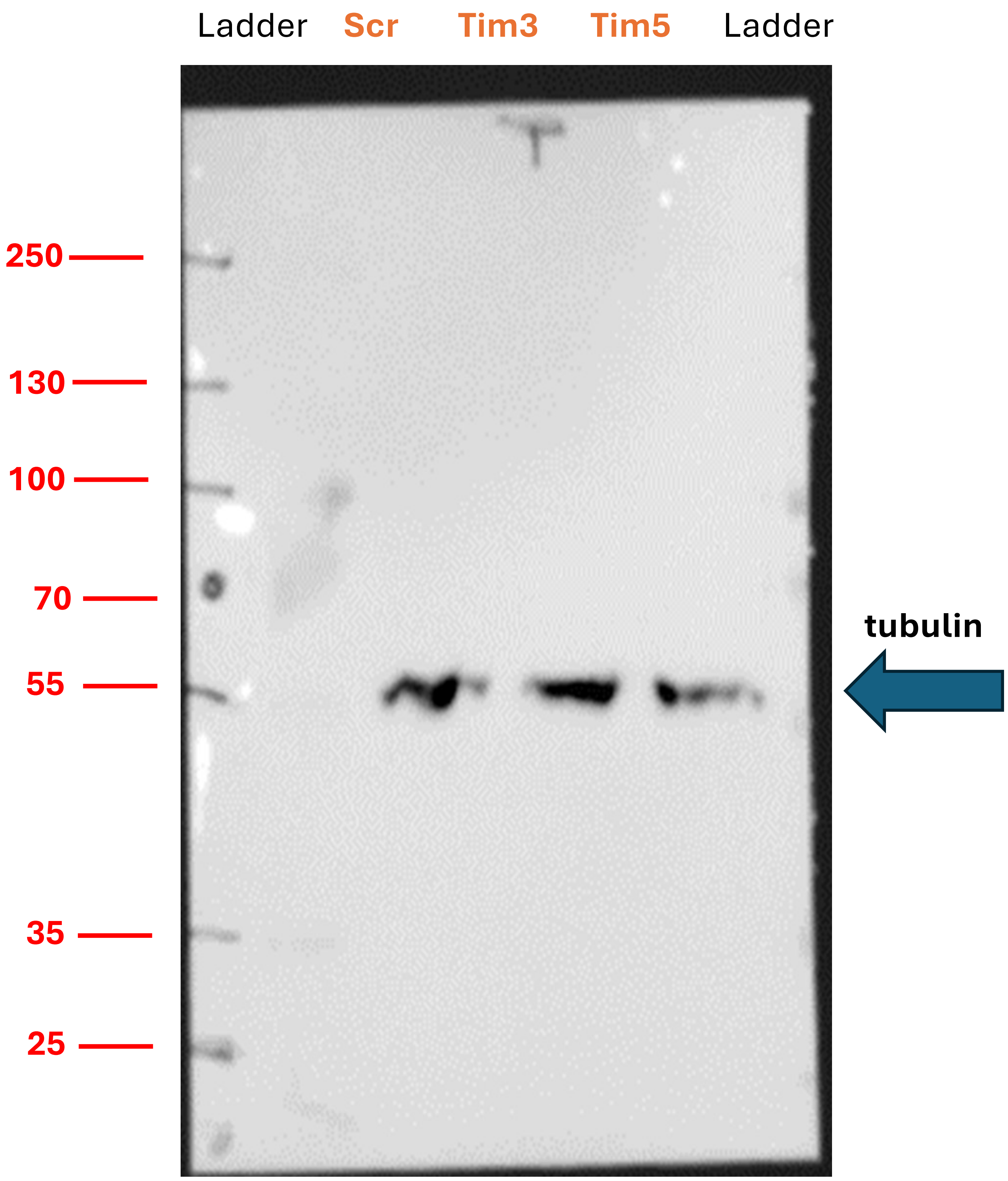

Uncropped Western blot images for Figure 6A

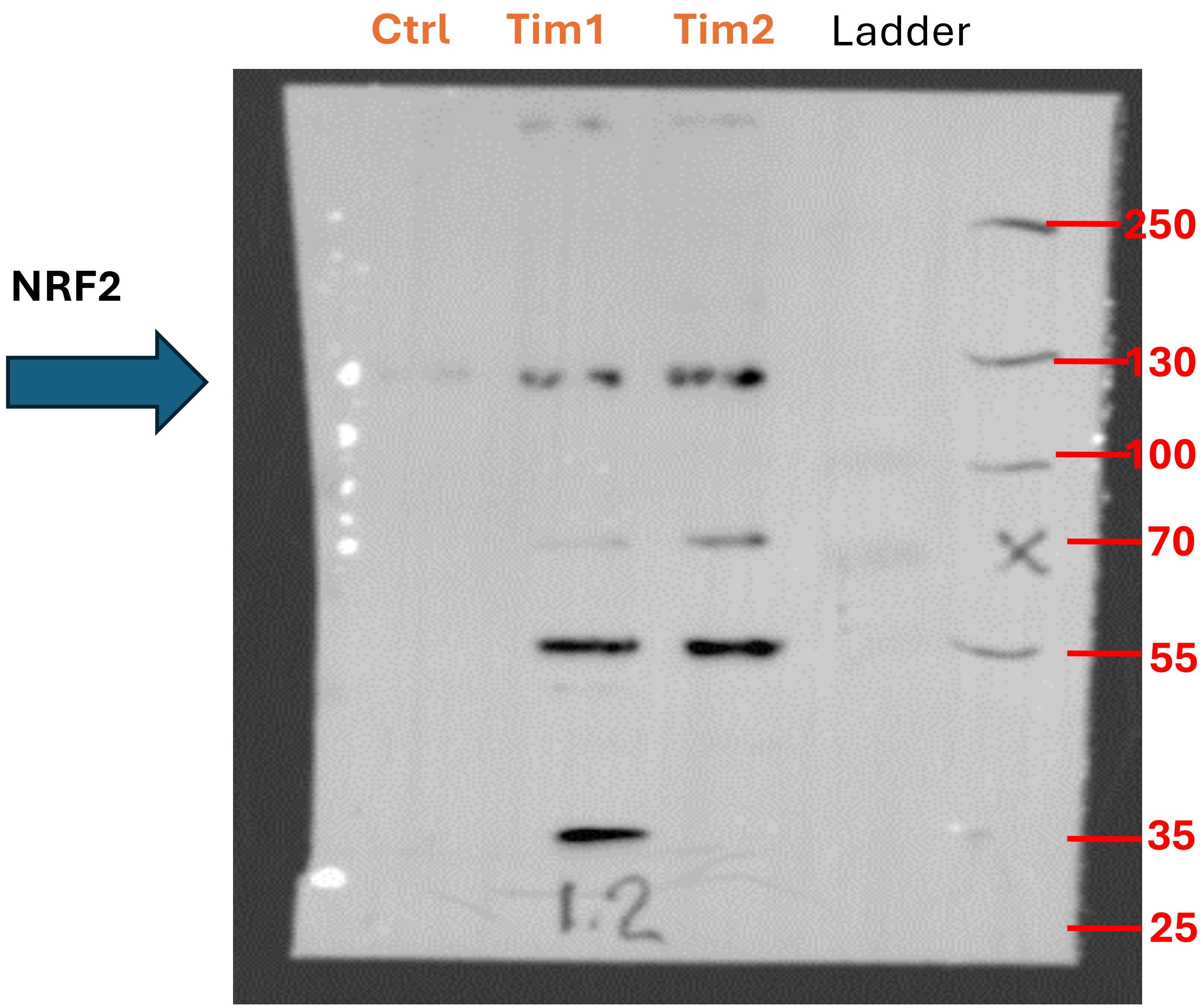

NRF2 intensity ratios: 0.04062 0.270247 0.433662

Uncropped Western blot images for Figure 6A

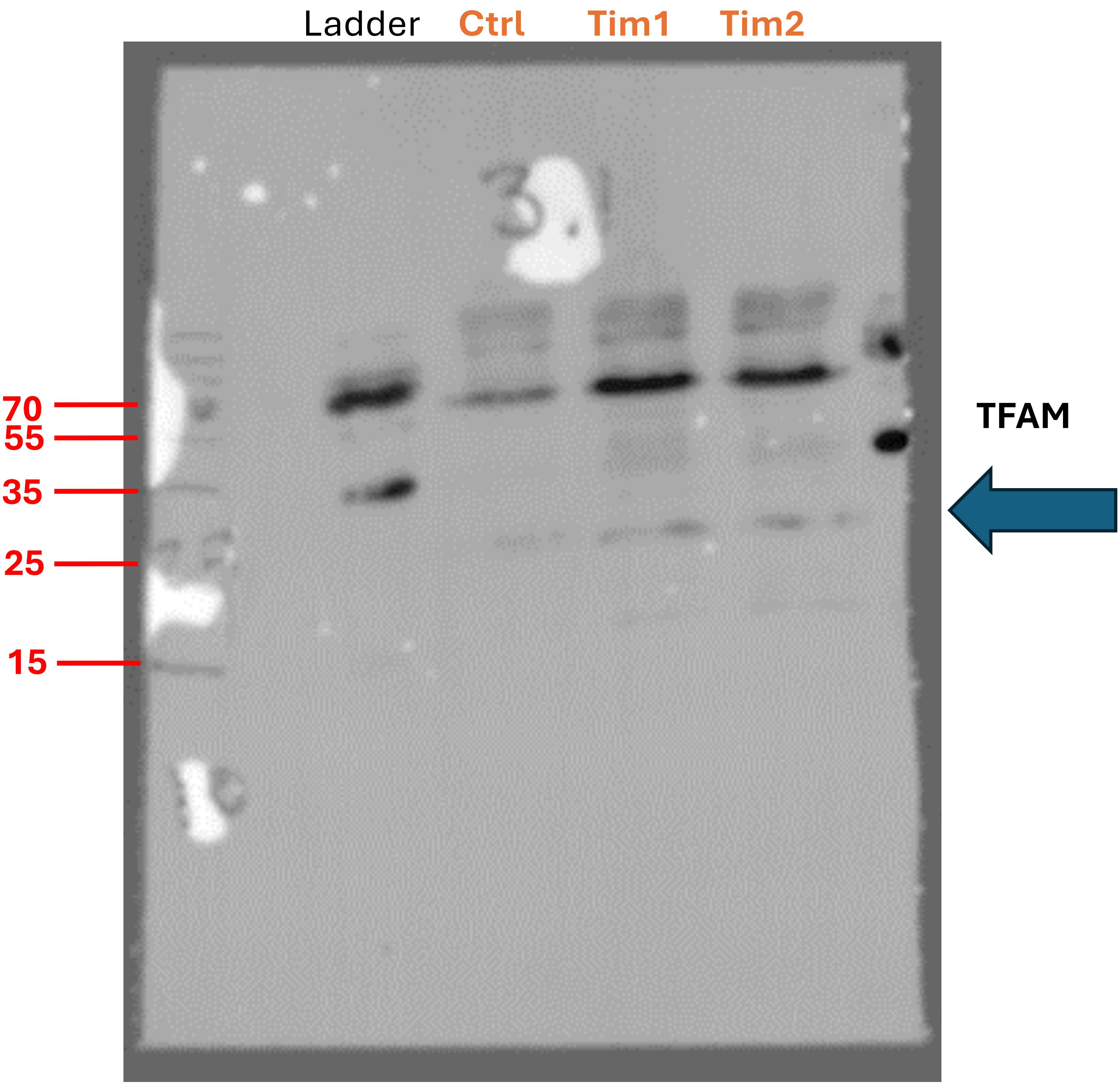

TFAM intensity ratios: 0.036189, 0.095482, 0.08188

Uncropped Western blot images for Figure 6A

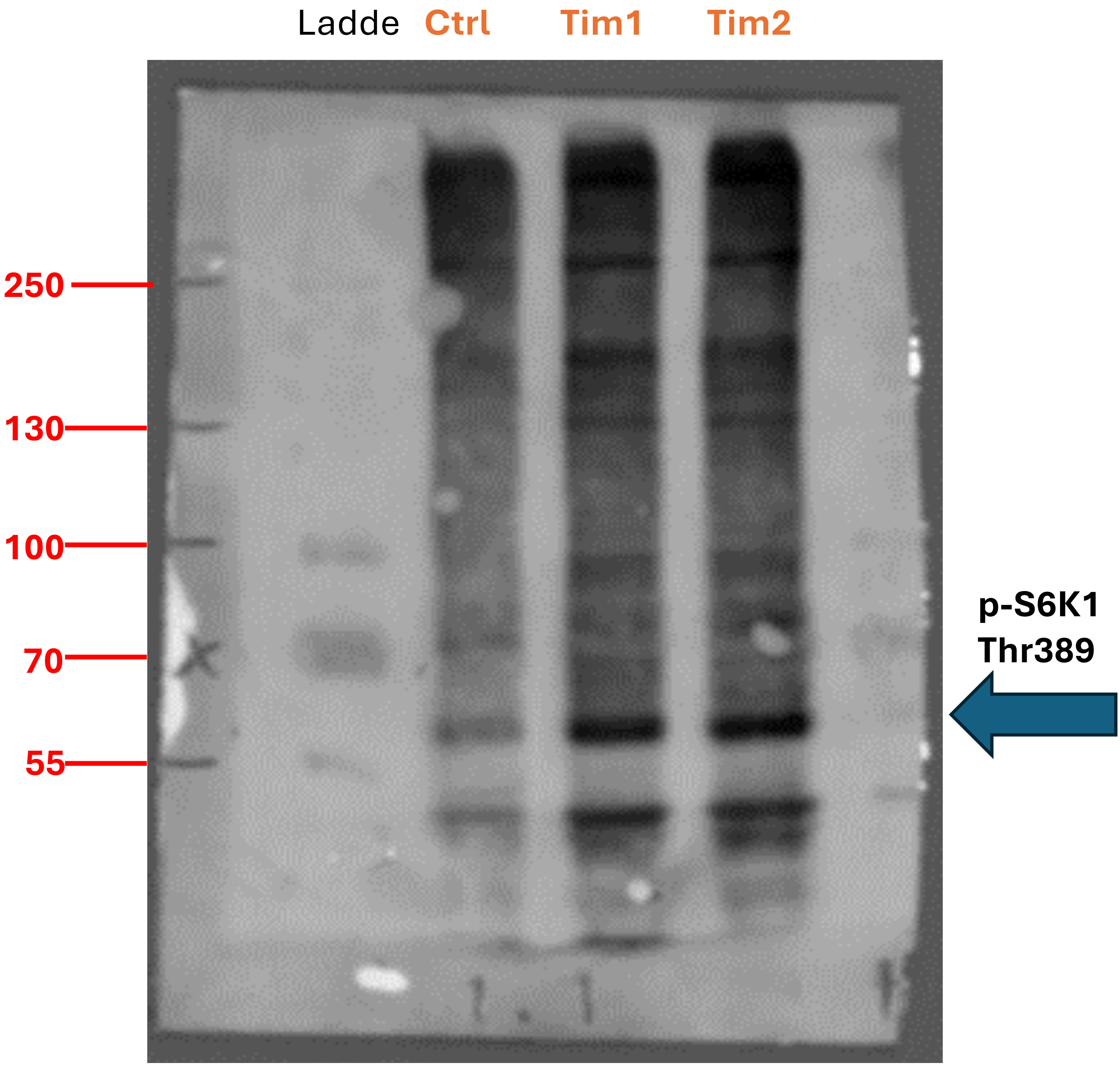

p-S6K1 intensity ratios: 0.223043, 0.612106, 0.522365
